# Connecting the organizational incomes and outcomes: a systematic review of the relationship between talent management, employee engagement, and turnover intention

**DOI:** 10.3389/fpsyg.2024.1439127

**Published:** 2024-07-10

**Authors:** Luna Sinisterra, Jonathan Peñalver, Marisa Salanova

**Affiliations:** ^1^Equipo de investigación WANT, Universitat Jaume I, Castelló de la Plana, Spain; ^2^Universidad Internacional de Valencia, Valencia, Spain

**Keywords:** talent management, employee engagement, turnover intention, systematic review, work engagement (WE), organization engagement

## Abstract

**Introduction:**

In a post-pandemic environment, characterized by volatility and uncertainty, organizations need to adapt to it for their survival.

**Methods:**

Following a systematic review method, the aim of this study is to assess the relationship between talent management practices, employee engagement, and turnover intention. Carried out using PRISMA guidelines, this systematic review includes 43 studies.

**Results:**

Results showed a lack of consensus on the talent management concept, definition, and measurement. Also, talent management practices seem to increase employee engagement and decrease turnover intentions. That is, when organizations provide effective talent management practices to employees, they become more engaged and less likely to abandon the company. It is important to highlight the mediating role of engagement in the relation between talent management and turnover intention. Furthermore, the most studied talent management practices for promoting engagement and reducing turnover intention were identified. Regarding control variables, data highlighted the importance of age and organizational tenure in the aforementioned relationships.

**Discussion:**

This review draws attention to the need of designing and implementing talent management practices in an effective way in order to generate a healthy and engaged workforce that is willing to remain in an organization.

## Introduction

The economic and work environment of the 21st century is highly dynamic and uncertain. In order to survive and succeed, organizations need to effectively adapt to changes in this environment (Luna-Arocas and Danvila-del-Valle, 2022). From 2020, two new phenomena have been added to this BANI (Brittle, Anxious, Nonlinear, and Incomprehensible) context: the COVID-19 pandemic and the subsequent “Great Resignation”, which stands for the situation in which a great number of employees voluntarily quit their jobs (Serenko, 2023). Working during the pandemic affected people's affective, cognitive, and behavioral processes, and their life and work priorities changed (e.g., working remotely, prioritize the psychological contract, live and work close to their family and friends, importance to work-life balance, etc.) (Malmendier, 2021; Serenko, 2023). This resulted in a shortage of talent that hampered organizations' strategies and operations (Formica and Sfodera, 2022; Tessema et al., 2022). Although in 2023 the Great Resignation ended and the quits slowed, it is logical for organizations to be afraid that a similar phenomenon may happen again, especially in the changing and uncertain environment we currently live in. Therefore, they want to be prepared to face upcoming similar situations.

In this context, the importance of Talent Management (TM) for organizations worldwide becomes evident. The ability not only to attract and develop talent, but also to retain it, is a challenge that all organizations are currently facing (Luna-Arocas and Danvila-del-Valle, 2022). However, despite its relevance in the organizational context, there is no consensus on the definition of talent management and the activities that comprise it (Dalal and Akdere, 2023).

In the uncertain environment that organizations face, TM practices are effective in reducing employees' turnover intention, and one of the mechanisms through which this relationship works is the mediating role of employee engagement (Fahmi et al., 2020; Kossyva et al., 2021; Alhajaj and Ahmad, 2023). Literature has studied the relationships among these variables for years, and there are various theoretical models explaining them, such as the HERO model (Salanova et al., 2012). However, five literature gaps have been identified.

First, although there are systematic reviews covering the concept of talent management itself or talent management with work engagement or turnover intention, there are no reviews integrating these three concepts along with the approach to their relationship. Second, even though the strong correlation between TM, employee engagement, and turnover intention has been demonstrated, together with the importance its effective management has in present times for the success of organizations and the wellbeing of workers, there are no systematic reviews that encompass the relationship between the three of them. Third, although some authors refer to engagement as a multidimensional concept formed of work engagement and organizational engagement—that is, as employee engagement (Saks, 2006),—reviews have only considered the work engagement approach, and not the organizational engagement one, which becomes highly relevant within the scope of this study. Fourth, as previously stated, there is no consensus on what talent management is and which activities it includes. Finally, the use of control variables results crucial in organizational research due to difficulties in the implementation of experimental and quasi-experimental design within this discipline (Bernerth and Aguinis, 2016). However, since there are no systematic reviews that link TM practices, engagement, and intention to quit, there is no consensual knowledge about which control variables are the most adequate to use when studying relationships between these three variables.

Therefore, the objective of this study is to address these gaps in literature by conducting a systematic review about talent management and its relationship with work and organizational engagement, as well as with employee turnover intention. It is our hope that the findings will not only guide future talent management and turnover intention research, but also show organizations how talent management practices and interventions can boost both work and organizational engagement, as well as retention of employees; and to broaden research in the field of organizational psychology.

## Theoretical background

### Talent management conceptualization

The concept of talent management has been gaining relevance and awareness since the early 2000s, and is becoming a process in continuous evolution (Gallardo-Gallardo et al., 2019; Sandeepanie et al., 2024). Despite this growth in its popularity, there is a lack of consensus on its definition (Dalal and Akdere, 2023). Although TM definitions may seem similar, currently, a huge number of definitions and frameworks coexist in research. Whereas, there are authors that defend talent management is a new and innovative concept, others expose talent management to be another way to refer to Human Resource Management (HRM), both including similar activities. In addition, there is a group of authors that defend TM covers the same activities as HRM but focusing on “talented people” rather than people in general (Sandeepanie et al., 2024). Despite this lack of consensus, there is one statement that is broadly accepted, namely that talent management is not a linear process but rather a cyclical one, composed of six stages: talent planning, talent identification, talent attraction, talent acquisition, talent development, talent deployment and talent retention (Yildiz and Esmer, 2023), which covers issues like selection, training, compensation, performance management, etc. (Pandita and Ray, 2018; Cajander and Reiman, 2024).

### Impact of talent management on employee engagement and turnover intention

Talent management has an important influence in the success and the financial results of an organization, since it improves productivity, job satisfaction, motivation, and organizational commitment; and reduces turnover intention (Chaudhuri, 2020; Kumar, 2022). TM strategies are effective in developing positive attitudes and, consequently, increase the desire of employees to stay in an organization and—accordingly—not to quit (Aburumman et al., 2020; Dayeh and Farmanesh, 2021).

Turnover intention is the degree in which an employee demonstrates willingness to finish his or her employment with the present employer (Triningsih and Darma, 2024). Previous studies identified variables such as talent management and human resource management practices (e.g., career development, performance management, rewards and recognition) as antecedents of employee turnover intention (Juhdi et al., 2013; Plessis et al., 2015). Additionally, an important concept in predicting and reducing employees' intention to quit is employee engagement (Bailey et al., 2017). According to previous studies, employees with low engagement levels result in higher turnover rates, while engaged employees show lower turnover rates (Vermooten et al., 2019).

Work engagement (WE) can be defined as “a positive, fulfilling, work-related state of mind that is characterized by vigor, dedication, and absorption” (Schaufeli et al., 2002, p. 74). Researchers have mainly focused on employee engagement as work engagement, being the previous definition the most accepted worldwide (Saks et al., 2022). However, Saks (2006) considers employee engagement (EE) as a multidimensional concept, formed by work engagement and organizational engagement (OE). Organizational engagement is defined as being highly positive about the organization, firmly connected to it, and willing to contribute to its success (Saks, 2006; Farndale et al., 2014). From now on, this study will refer to employee engagement when talking about both work engagement and organizational engagement; and will refer to them individually when studies only refer to one of them. Antecedents of employee engagement include job and personal resources (Saks, 2019). Moreover, research has shown that, in order to enhance work engagement, it is preferable to increase resources rather than reduce demands (Salanova et al., 2019). TM practices can be considered as job resources, thus, as antecedents of work engagement (Memon et al., 2021). Additionally, different studies show that talent management has a positive impact toward employee engagement (Alias et al., 2014; Aljunaibi, 2014; Abdullahi et al., 2022; Ekhsan et al., 2023). Among the consequences of employee engagement, positive attitudes such as higher job satisfaction, organizational commitment, and lower turnover intention stand out (Bakker and Demerouti, 2017; Saks, 2019). Research has demonstrated the positive influence that employee engagement exerts on employees' intention to stay (Shah and Beh, 2016; BowenXue et al., 2024). Therefore, in order to retain talent, companies should focus on how to engage their employees (Aktar and Pangil, 2018; Sheikh et al., 2020).

### The HERO model: a framework to explain these relationships

**Figure 1:**
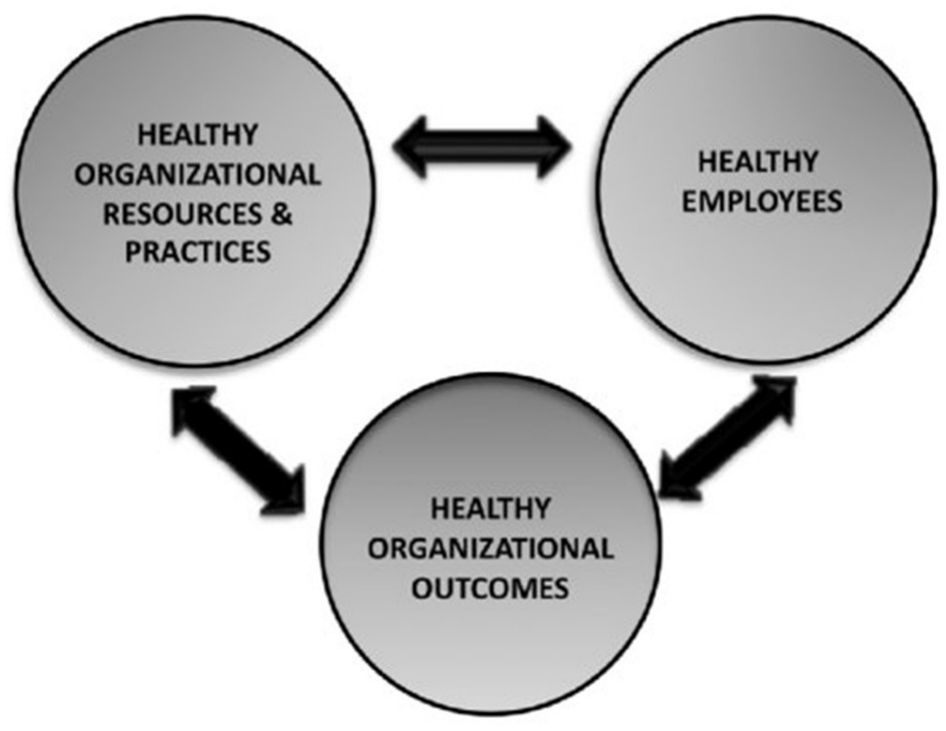
HERO model.

One theoretical framework that illustrates the aforementioned relationships is the HERO model developed by the WANT Research Team. The HERO model conforms a heuristic and theoretical framework of healthy and resilient organizations. According to this model, a healthy organization is comprised of three interrelated elements: (1) Healthy Organizational Resources and Practices, (2) Healthy Employees, and (3) Healthy Organizational Results (Salanova et al., 2012). For the purpose of this study, talent management practices are considered within the first dimension (Healthy Organizational Resources and Practices), employee engagement is part of the Healthy Employees dimension, and turnover intention belongs to the third dimension of the model (Healthy Organizational Results). Consequently, a clear interplay between variables can be observed, being employee engagement in the middle, as a mediator (Figure 1).

As stated in the model, these three concepts are interrelated. Retention of employees is becoming crucial in recent times. Moreover, talent management is decisive for promoting employee engagement which, at the same time, is determining for employee retention. During and after the COVID-19 pandemic, people experienced low levels of employee engagement and great levels of turnover intention (Shukla et al., 2022; Lee et al., 2023). In addition, although the “Great Resignation” is over, employees still aspire to get better jobs with better conditions or in more desirable industries (Morgan, 2023). Therefore, it is crucial for organizations to understand how to increase employee engagement and reduce their desire to abandon the organization. From a positive perspective, the COVID-19 pandemic and the “Great Resignation” can be seen as an opportunity for organizations to reform their talent management strategies and prepare for future events (Hu et al., 2023).

Given the objective of this study, the identified literature gaps and the existing research on talent management, employee engagement and turnover intention, this systematic review will address the following research questions:

*Research Question 1:* What is talent management? How is talent management measured? What theories do researchers use in order to address the effect of talent management on engagement and turnover intention?
*Research Question 2:* Which talent management activities are studied most recurrently in research regarding their relation with employee engagement and turnover intention?
*Research Question 3:* How is the relation between talent management practices, employee engagement, and turnover intention?
*Research Question 4:* Which control variables are taken into account in the relationship between talent management practices, employee engagement, and turnover intention?

## Method

### Search strategy

The study was carried out following PRISMA guidelines (Moher et al., 2009). A search string that matched the inclusion criteria was constructed (see Table 1). The search was performed in February 2023 in four commonly used databases (Scopus, Web of Science, Business Source Premier, and ProQuest) to get good search coverage when conducting literature reviews (Lam and McDiarmid, 2016). These databases were selected because they include multiple disciplines and contexts (Scopus, Web of Science, and ProQuest) and cover aspects and constructs of the business and management field (Business Source Premier). An “Only abstract” filter was used in the database Business Source Premier, and an “All except whole text” filter was used in ProQuest since, if not, results would have been countless and irrelevant. In the remaining databases, no filters were used. In the databases that allowed it (Business Source Premier and ProQuest), research was limited to peer-reviewed articles. There were no limitations regarding publication year. In May 2024, a new search was conducted to update the systematic review with the studies published during 2023 and 2024. The search string and procedure were the same as in the initial search, but the publication years were limited to 2023 and 2024.

**Table 1:** Search string.

**Search criteria**	**Search string**
Complete search string	(engagement OR “work engagement” OR “job engagement” OR “organizational engagement” OR “organisational engagement” OR “employee engagement” OR “task engagement”) AND (“talent management” OR “talent management practice^*^” OR “talent management intervention^*^” OR “human resource management” OR “human resource management practice^*^” OR HRM OR “human resource practice^*^” OR “human resource system” OR “human resource management system”) AND (“intention to quit” OR “turnover intention” OR abandon^*^ OR “intention to abandon” OR “intention to leave” OR rotation)
a. Engagement	(engagement OR “work engagement” OR “job engagement” OR “organizational engagement” OR “organisational engagement” OR “employee engagement” OR “task engagement”)
b. Talent management	(“talent management” OR “talent management practice^*^” OR “talent management intervention^*^” OR “human resource management” OR “human resource management practice^*^” OR HRM OR “human resource practice^*^” OR “human resource system” OR “human resource management system”)
c. Intention to quit	(“intention to quit” OR “turnover intention” OR abandon^*^ OR “intention to abandon” OR “intention to leave” OR rotation)

### Inclusion criteria

The inclusion criteria were:

1. The article describes an original empirical quantitative research study. Qualitative studies and case studies were not considered. This review only includes quantitative studies since quantitative data facilitates the comparison and synthesis of results across studies and help to reduce biases from research, providing a better consistency and accuracy when analyzing relationships between variables.
2. The article is written and published in English or Spanish (corresponding to language proficiencies among authors).
3. The study was conducted in an organizational environment, either public or private.
4. The study addresses talent management issues and practices. Concretely, only the following five practices will be taken into consideration as TM practices, since (1) the number of TM activities can be countless and, therefore, the development of this review would be struggling and non-concise; and, more importantly, (2) according to research, these five activities are the most broadly accepted as TM activities in relation to employee engagement and turnover intention (Pandita and Ray, 2018): selection and recruitment, training and development, performance management, rewards and recognition, and career development. The selection of these five practices has been done according to existing frameworks of talent management in the literature, which point out these five activities as being the most representative of the talent management function (Bolander et al., 2017; van Zyl et al., 2017). Hence, this study narrows the scope of TM down to five activities in order to get a better and more concise understanding of their relationship with employee engagement and turnover intention. Additionally, in order make sure this review covers every perspective and approach of TM, there have been included studies addressing Human Resource Management, High-Performance Human Resource Systems and other terms that include at least one of the five TM practices considered in this review. As previously mentioned, there are authors who consider TM to be the same as these other terms. Moreover, in both academic and business contexts these terms are often used interchangeably, and TM activities are often integrated into the human resource function. Therefore, in order to offer a comprehensive and representative literature review in this field, papers studying TM activities included under these terms will be included.
5. The article presents data about the relationship between talent management practices, engagement, and turnover intention.

### Study selection and data extraction

After the initial search of records and the removal of duplicates, one author screened the titles, abstracts, and keywords to identify potentially relevant studies. Studies were included or rejected based on language, type of study, topics addressed, and measures included. Studies that might meet the inclusion criteria were included.

**Figure 2:**
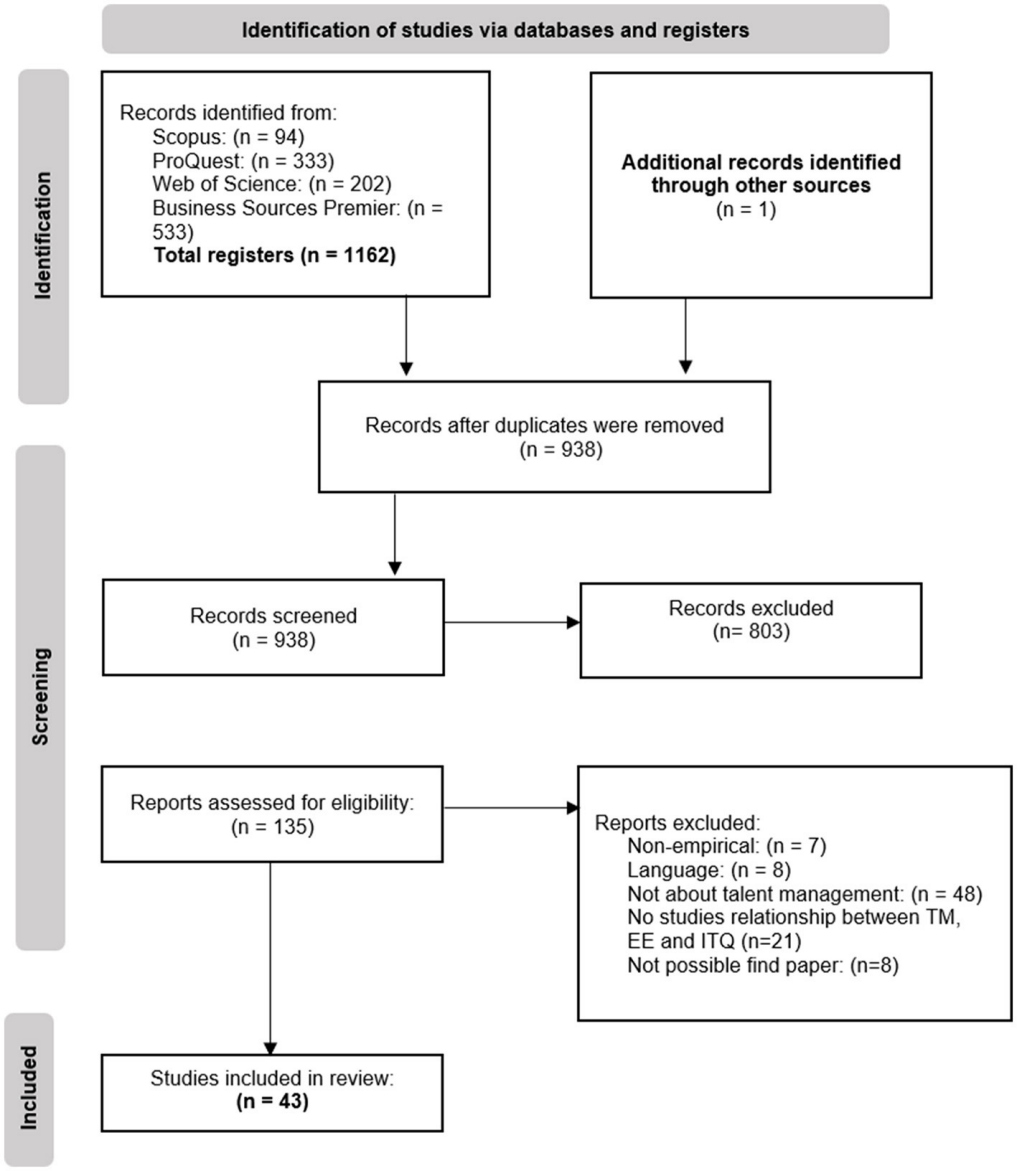
PRISMA flow-chart.

The following phase included a full article review to identify articles that met all the inclusion criteria. Two authors analyzed the full text of records that were assessed as eligible, based on their abstracts. Finally, articles that met the inclusion criteria were included in the review. A PRISMA (Preferred Reporting Items for Systematic Review and Meta-Analyses) flow-chart is included for reference (Figure 2).

After identifying the studies to be included, data extraction was conducted. Authors extracted demographic and outcome data (location, research design, sample, sector, main variables, definitions, and results) from the studies included. Discussions concerning the data extraction were held between authors to ensure consistency. Authors reviewed all data extractions for completeness and accuracy.

## Results

The search strategy resulted in retrieving 1,065 articles in February 2023. Additionally, the search strategy conducted in May 2024 to update the systematic review resulted in 97 studies. Moreover, one article was identified through other sources. Therefore, the search yielded a total of 1.163 studies. Yielded articles were published from the inception of the data up to 20th May 2024. After duplicates were removed (*n* = 225), 938 articles remained for the screening of titles and abstracts. The complete review of articles was conducted for 135 papers, of which 92 were discarded because they did not meet the inclusion criteria for the following reasons: they were not quantitative empirical studies (*n* = 7); they were in a different language than English or Spanish (*n* = 8); the content was not about talent management practices (*n* = 48); they did not analyze the relationship between talent management practices, employee engagement, and turnover intention (*n* = 21); or it was not possible to access the paper (*n* = 8). Regarding talent management practices, as pointed out in the introduction, only studies covering the following practices have been included in this review: selection and recruitment, training and development, performance management, rewards and recognition, and career development. Papers studying support for participating in these activities were not included in this research, since they did not study the practice itself or the perception of the practice, but rather the support received to participate in these activities. Eventually, 43 articles met the inclusion criteria and were included in the review. It is important to note that relevant missing information has been requested to authors through email or the ResearchGate website.

### Study characteristics

Studies were conducted in 25 **countries** on all five continents. Among these countries, Malaysia and India stand out, with 18.6% (*n* = 8) and 9.3% (*n* = 4) of the studies conducted in each, respectively. For more information about where the studies were conducted, see Supplementary Table S1.

In regard to the **sector** were the participants of the study worked, 35% (*n* = 15) of the studies included were conducted in multiple sectors; 14% (*n* = 6) were conducted in the services sector; 11.6% (*n* = 5) of the studies were conducted in the health sector; and 11.6% (*n* = 5) in the industrial sector. For information about the sectors covered in the remaining studies, see Supplementary Table S1.

Finally, regarding **research design and methodology**, all articles (*n* = 43) were empirical and used a quantitative methodology (e.g., survey method). Among them, 93% (*n* = 40) were cross-sectional studies and 7% (*n* = 3) were longitudinal time-lagged studies. In addition, 11.6% (*n* = 5) of the articles adopted a multilevel approach.

**Research Question 1:** What is talent management? How is talent management measured? What theories do researchers use in order to address the effect of talent management on engagement and turnover intention?

As mentioned previously, the studies included addressed at least one of the following TM practices: selecting and recruiting, training and development, performance management, rewards and recognition, and career development (Pandita and Ray, 2018). However, it has been found that each study gives a different name or definition to this bundle of practices. First, results will address the issue about the concept of talent management. Names and terms of TM practices of each article are available in Supplementary Table S2. One of the most used terms, rather than talent management, was High-Performance Work Systems (HPWS) (*n* = 10), in which both high-performance work systems and high-performance human resource practices were included. Definitions of this concept vary between articles and studies. The study of Kloutsiniotis and Mihail (2017, p. 35) defined it as “a specific combination of HR practices, work structures, and processes that maximizes employee knowledge, skill, commitment, and flexibility”. de Oliveira and da Silva (2015, p. 1023) defined HPWS as “a bundle of human resource management (HRM) policies and practices designed to create a more productive workforce, therefore adding value to the organization and its internal customers”. A study from Finland defined them as a bundle of interrelated practices implemented toward employees in order to achieve superior organizational outcomes (Ehrnrooth et al., 2021).

Another broadly used concept to refer to this set of practices is Human Resource Management Practices (*n* = 10), which are defined by Memon et al. (2021) as a group of individual but internally consistent practices aimed to promote an organization's human capital in accordance to its organizational employees. On the other hand, Juhdi et al. (2013) define them as the ways an organization uses to shape employee's behaviors and attitudes. Kossyva et al. (2024) define them as a formal and structured process of an organization in order to attract, develop and retain talent that helps the organization to gain a competitive advantage.

Continuing with naming and nomenclature, four studies (*n* = 4) talk about talent management or TM practices. One study defines it as “the systematic attraction, identification, development, engagement/retention, and deployment of those individuals with high potential who are of particular value to an organization” (Bui and Chang, 2018, p. 3). This definition is aligned with the one provided by Fahmi et al. (2020) who conceptualized it as a process that focuses only on those individuals who can provide competitive advantage to a company by managing these people in an effective and efficient way. On the other hand, Rumawas (2021) considers talent management as a group of long-term organizational strategies to achieve a competitive advantage by placing the right people in the right place at the right time.

The remaining studies conceptualize the object of this review as job resources (*n* = 2), antecedents of engagement (*n* = 1), human resource development (*n* = 2), high-involvement human resource practices (*n* = 1), green human resource management (n = 1), age-diversity practices (*n* = 1), employee-friendly company (*n* = 1), and psychological contract (*n* = 1). Twenty-two percentage (*n* = 9) of the studies did not conceptualize the object of the study in any way, since they examined isolated practices.

Second, there is no consensus regarding the way in which talent management and TM practices are measured, since each study used a different scale. The majority of the TM practices questionnaires are self-constructed, using information from different authors. Further information about scales used to measure study variables is presented in Supplementary Table S3.

Third, in regard to psychological theories used to address the relationships among TM practices, engagement, and turnover intention, 60% of the studies (*n* = 26) rely on the Social Exchange Theory (SET) (Homans, 1961; Blau, 2017) to explain these relationships. According to Saks (2006), employees decide how much they engage in their job and organization derived from the resources they perceive from them. The SET defends that positive work outcomes (such as employee engagement and intention to stay) are ways of reciprocation by employees to organizations' practices and systems. Organizational practices and policies show employees that the organization is committed to their personal and professional wellbeing, which creates a moral obligation in employees to respond to these efforts of the organization by enhancing their work efforts and performance (Alfes et al., 2013; Kakkar et al., 2020). Otoo (2022), using SET, defends that each party in a relationship has a mutual duty toward the other, and that the assessment of benefits and costs impact on how people interact with each other.

Moreover, 23% of the studies (*n* = 10) based their hypothesis on the JD-R model. The JD-R model (Schaufeli and Bakker, 2004) presents two psychological processes in the relation between demands, resources, and wellbeing: (1) the energetic process that links job demands with burnout and—consequently—with health problems, and on which job resources have a negative relationship with burnout and illness; and (2) the motivational process that links job resources with work engagement and organizational outcomes, and on which job resources have a positive relationship with work engagement and—consequently—with health and performance (Schaufeli and Bakker, 2004). Talent management practices can lead to an increase in job and personal resources, thus promoting work engagement and reducing turnover intentions (Lee et al., 2019; Kakkar et al., 2020).

Other theories such as the equity theory, the leader-member exchange theory, or the AMO framework are also mentioned in different articles.

**Research Question 2:** Which talent management practices are studied most recurrently in research regarding their relation with employee engagement and turnover intention?

This question addresses the matter of finding which are the most studied TM activities in research regarding their influence on employee engagement and turnover intention. As mentioned earlier, in this study, only five talent management practices have been considered for being the most popular ones in research. In general, all five practices were broadly studied in the articles included in this review. More concisely, the most studied practice for its relation with engagement and turnover intention was Rewards and recognition. Rewards and recognition refers to the distribution of tangibles and intangibles to employees in exchange for good performance (Babakus et al., 2017). As defended by Saks (2006) and Alferaih et al. (2018), among others, employees will be more likely to engage at work and stay in an organization if they perceive a satisfactory number of rewards and recognition for their performance. This practice is followed by Performance management, Training and development, Career development and, finally, Selection and recruitment. Performance management refers to the system for establishing goals, providing feedback, giving appraisal, and supplying recognition to employees based on their performance (Kakkar et al., 2020). According to the Social Exchange Theory, employees are likely to identify an effective performance management system as an indicator of the organization's commitment toward their development, and respond by making refinements to their behavior, thus increasing engagement and reducing turnover intentions (Kakkar et al., 2020). Training and development refers to a set of planned activities in order to promote job knowledge and skills, or to alter attitudes and social behavior of workers aligned with organizational goals and job requirements (Memon et al., 2016). Based on the Social Exchange Theory, research has found evidence that training and development promotes engagement (Kloutsiniotis and Mihail, 2017) and reduces turnover intention (Memon et al., 2016). Career development refers to “the planning, guiding and developing employees' careers in the organization” (Marescaux et al., 2012, p. 8). Its objective is to aid employees in their career development, growth, and learning in a way that benefits both the employee and the organization (van der Merwe et al., 2020). Therefore, when employees feel their organization cares about their career growth and development, they will feel their organization cares for them and values their development, promoting engagement and reducing the intention to quit (Sheehan et al., 2019; van der Merwe et al., 2020). To sum up, selection and recruitment refers to “the process of attracting, selecting and retaining competent individuals to achieve organizational goals” (Anjum and Din, 2022, p. 3). An effective selection process will result in higher engagement and lower turnover intentions, since employees' abilities will match the job requirements (Juhdi et al., 2013; Gadi and Kee, 2020).

**Research Question 3:** How is the relation between talent management practices, employee engagement, and turnover intention?

**Figure 3:**
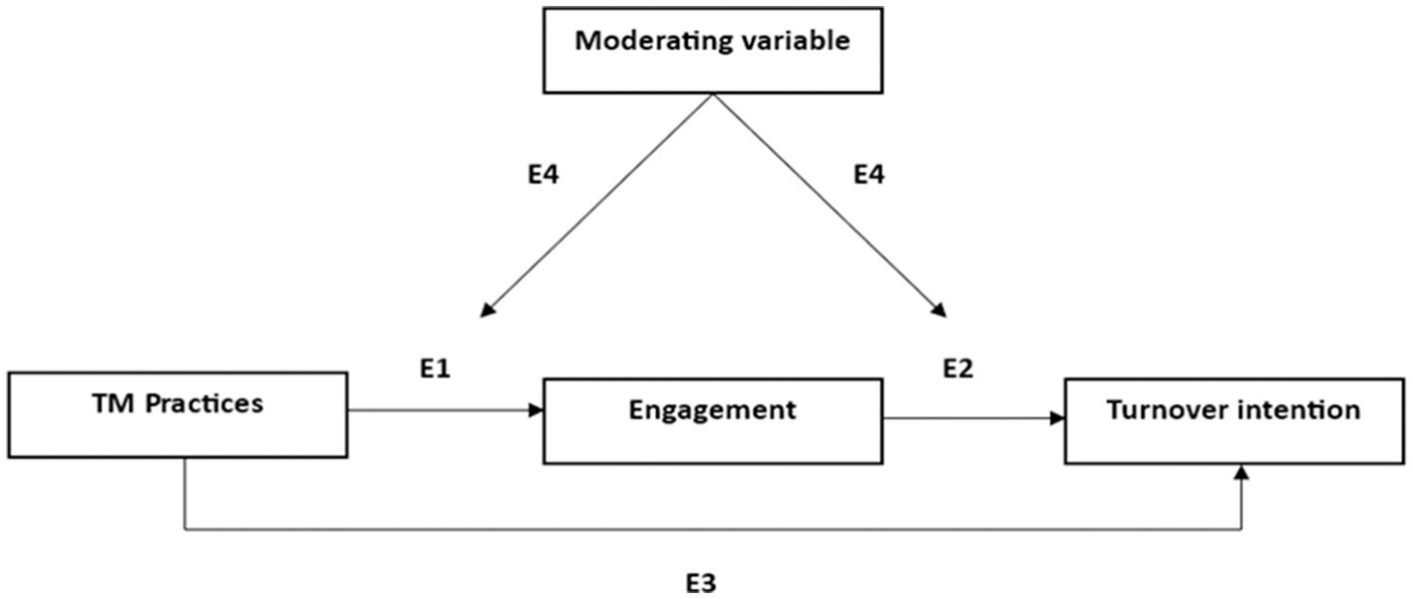
Effects analyzed in the model.

In order to answer this question, data has been classified into four categories: (1) direct effect of TM practices on employee engagement; (2) direct effect of TM practices on turnover intention; (3) mediation effects; and (4) moderating effects (see Supplementary Table S4 for more details). Figure 3 shows the proposed model for studying these relationships, each number corresponding to one of the section categories.

1. *Direct effect of TM practices on work or organizational engagement (E1):* 89% (*n* = 35) of the studies that measured this relationship (*n* = 39) reported a significant positive effect of TM practices on work or organizational engagement. Nevertheless, some of these studies showed practices that do not report a significant association with work or organizational engagement. For example, some authors found a non-significant relation between rewards and recognition and work engagement, while this relationship was significant with performance appraisal and training (Memon et al., 2021), as well as with career development (van der Merwe et al., 2020). Moreover, Bui and Chang (2018) found soft TM practices (training and development) to exert a non-significant effect on employee engagement, while this effect was significant for hard TM practices (selection and recruitment, career development, rewards and recognition, performance management). On the other hand, a study from Malaysia found career development to be the strongest predictor of organizational engagement (Juhdi et al., 2013). There is one article (Ang et al., 2013) that yielded different results for different sample groups. On the contrary, three articles showed non-significant relations between all TM practices and work engagement (Babakus et al., 2017; Sheehan et al., 2019; Islam et al., 2023), and one article reported a non-significant relation between TM practices and employee engagement (Saks, 2006). Furthermore, four articles did not measure this relationship (Shah and Beh, 2016; Katou, 2017; Alferaih et al., 2018; Rezwan and Takahashi, 2022). It is also important to highlight that only 23% (*n* = 10) of the studies considered the organizational approach of engagement. The remaining studies (*n* = 33) only took into account work engagement.
2. *Direct effect of TM practices on turnover intention (E2):* 89% (*n* = 25) of the studies that measured this relationship (*n* = 28) reported a significant negative effect of TM practices on turnover intention, supporting the hypotheses of the studies. However, some of these articles showed practices that do not report significant association with turnover intention. For instance, two studies found rewards and recognition do not influence turnover intention, while they found a significant negative effect of selection and recruitment, training and development, performance appraisal and career development, on turnover intention (van der Merwe et al., 2020; Ramaprasad et al., 2021). Otherwise, Babakus et al. (2017) reported a negative significant relationship between rewards and turnover intention, but a non-significant relationship between training and development and turnover intention. On the other hand, Juhdi et al. (2013) found that career development had a non-significant effect on turnover intention, whereas rewards and recognition, as well as performance appraisal, had a significant negative effect on turnover intention. Finally, a study from Vietnam reported significant negative effects of hard TM practices (selection and recruitment, career development, rewards and recognition, performance management) on turnover intention, but a non-significant relation between soft TM practices (training and development) and turnover intention (Bui and Chang, 2018). Following this category of results, three studies (Sheehan et al., 2019; Sousa et al., 2021; Winarno et al., 2022) did not find the relation between any of the TM practices and turnover intention to be significant. The remaining fifteen studies did not measure this relationship.
3. *Mediating effects (E3):* this section includes those articles that consider work or organizational engagement as a mediating variable between TM practices and turnover intention (*n* = 38; 88%). Specifically, 31 studies (81%) confirmed a significant mediation effect. However, in some of these studies, with regard to engagement as a mediating variable, data reported different results, since significant and non-significant relations were found, depending on which independent and dependent variables were tested. For instance, in some studies, engagement mediates the relationship between some TM practices and turnover intention, but when it comes to the practice “Rewards and recognition”, work engagement does not mediate this relationship (van der Merwe et al., 2020; Wen et al., 2022). Mismatched effects have been found in the studies of Shah and Beh (2016), where organizational engagement mediates the relationship between TM practices and intention to quit, but work engagement does not; and Bui and Chang (2018), where employee engagement mediates the relation between hard TM practices (selection and recruitment, career development, rewards and recognition, performance management) and turnover intention, but it does not mediate the relation between soft TM practices (training and development) and turnover intention. On the other hand, in two study (Sheehan et al., 2019; Islam et al., 2023), work engagement does not mediate the relationship of any of the TM practices and turnover intention. Finally, there are five articles (13%) that situate engagement in a mediating position, but the authors did not test this relation through appropriate analyses (Baron and Kenny, 1986; MacKinnon et al., 2002).
4. *Moderating effects (E4):* Considering only those studies that examined the effect of different moderating variables in the relationship between TM practices, work or organizational engagement and turnover intention (*n* = 11); results vary. Moderating variables have been classified for the purpose of this section as: (1) personal-level variables, (2) leader-level variables, (3) organizational-level variables, and (4) socio-demographic variables. With regard to (1) personal variables, work centrality moderates the relation between age-diversity practices and work engagement (Sousa et al., 2021). In addition, customer orientation moderates the relationship between TM practices and work engagement, and TM practices and turnover intention (Babakus et al., 2017). Therefore, customer orientation strengthens the positive relation of TM practices and work engagement, and the negative relation of TM practices and turnover intention. Moreover, functional competence is found to moderate the relationship between rewards and recognition and work engagement, but not the relationship training and development and work engagement (Islam et al., 2023). Finally, self-efficacy does not moderate the relationship between work engagement and turnover intention (Alhajaj and Ahmad, 2023). Regarding (2) leader-level variables, leader-member exchange quality was found not to moderate the relationship between employee engagement and turnover intention (Alfes et al., 2013). With regard to (3) organizational variables, a study from India revealed organizational culture not to moderate the relationship between TM practices and work engagement nor turnover intention (Dalal and Akdere, 2023). Moreover, perceived organizational support was found to moderate the relationship between employee engagement and turnover intention (Alfes et al., 2013; Yusliza et al., 2021). That is, the negative relationship between employee engagement and turnover intention was lower when perceived organizational support was low than when it was high (Yusliza et al., 2021). Furthermore, high-performance human resource practices were found not to moderate neither the relationship between proactive personality and work engagement or turnover intention (Rezwan and Takahashi, 2022), nor the relationship between transformational leadership and work engagement or turnover intention (Ehrnrooth et al., 2021). Finally, (4) socio-demographic variables such as position level and financial insecurity were taken into consideration. On the one hand, Wen et al. (2022) demonstrated that position level does not moderate the relation between TM practices and turnover intention. On the other hand, financial uncertainty was found to moderate the relationship between work engagement and turnover intention (Anjum and Din, 2022).

**Research Question 4:** Which control variables are taken into account in the relationship between talent management practices, employee engagement, and turnover intention?

As stated in the review of Bernerth and Aguinis (2016); gender, age, and tenure are the most used control variables in organizational research. Therefore, the same controls are expected to be the ones most used in the studies addressing these study's control variables.

In order to answer this question, it has been divided into two sections. First, control variables used in studies and the reason to include them will be identified. Among the articles included in the review, only 39% of the studies (*n* = 17) have analyzed control variables. Among them, the most used control variables were gender (*n* = 12), age (*n* = 13), and organization tenure (*n* = 7). Other control variables used in studies were sector (*n* = 5), position (*n* = 5), organization size (*n* = 4), work experience (*n* = 2), number of job offers (*n* = 1), education (*n* = 4), supervisor sex (*n* = 1), perceived organizational support (*n* = 1), permanent job or not (*n* = 2), full-time or part-time job (*n* = 1), trust in supervisor (*n* = 1), working hours (*n* = 2), tenure under the same supervisor (*n* = 1), setting of employment (*n* = 1), marital status (*n* = 1), and white or blue collar occupation (*n* = 1). The majority of the studies used these control variables only based on previous research (e.g., Marescaux et al., 2012; Lam and McDiarmid, 2016).

Second, the impact of control variables on the relationship between TM practices, employee engagement, and turnover intention will be analyzed. After examining correlations of the most commonly used control variables, it could be concluded that gender did not show a significant correlation with the study variables (TM practices, engagement, and turnover intention)—since it only correlates significantly with 15% of the articles in which gender is treated as a control variable—while age and organizational tenure appear to correlate significantly with study variables—they correlate significantly with 62 and 63% of the articles in which they appear, respectively. It is important to note that some articles did not contain information about correlations, which is why it has not been included. Therefore, if all information had been available, the results might have been different. See Supplementary Table S5 for more information.

## Discussion

The aim of this systematic review was to offer an integrated overview of the literature about talent management and its relation with work or organizational engagement and turnover intention. This was done with the objective of understanding how organizations can promote the desire to stay of their employees, an issue of special interest in today's world characterized by uncertain events, such as the COVID-19 pandemic. Therefore, 43 studies addressing these issues were found and analyzed in this systematic review. In an attempt to logically summarize the purpose, four research questions were proposed. Results and discussion have been summarized following these research questions. After all the obtained articles were reviewed, among other results, the most relevant ones show: a clear need to reach a consensus on talent management definition; the importance of effectively designing and implementing TM practices and strategies in order to retain employees; and the relevance of enhancing employee engagement in order to retain employees through TM practices. In this section, results are discussed following the research questions set out for the review. Moreover, theoretical and practical implications, study limitations, avenues for further research, and a future research agenda are identified.

**Research Question 1:** What is talent management? How is talent management measured? What theories do researchers use in order to address the effect of talent management on engagement and turnover intention?

First, literature builds on diverse conceptualizations and definitions of talent management. Results indicate a lack of consensus, not only on the definition of talent management, but also on its naming and categorization. Regarding the definition of talent management, we find two different categorizations of the concept. On the one hand, there are authors who define TM as a process that needs to be directed toward the whole organization in order to increase performance and positive employee outcomes (e.g., Marescaux et al., 2012; Oliveira and da Rocha, 2017). On the other hand, there is a tendency that considers TM practices need to be addressed only to those employees who stand out from the others and have the potential to drive the company toward success (e.g., Bui and Chang, 2018; Fahmi et al., 2020). This controversy yields to different studies addressing different topics but using the same terminology.

Second, in regard to the naming aspect, results have shown a huge number of names that are given to the same activities (see Supplementary Table S2). These concepts have slight differences in their definitions but, in the end, they all include the same five activities considered in this review, among others. This number of names and nomenclature is the reason why research—despite studying the same practices—is divided into different categories. Therefore, it is necessary to reach a consensus and aggregate practices under the same term to facilitate research and organizational actions. In this review, it is proposed to link the definition that considers focusing only on high-potential employees with the nomenclature “Talent Management” (Bui and Chang, 2018; Fahmi et al., 2020; Dalal and Akdere, 2023), and the tendency that considers focusing on every employee to fall under the name of “High-Performance Work Systems” or “Human Resource Management” (de Oliveira and da Silva, 2015; Shah and Beh, 2016; Katou, 2017; Gadi and Kee, 2020; Ehrnrooth et al., 2021; Kossyva et al., 2021). In that way, name and definition will be in line with research and literature, as this review's results show.

Third, there is also a lack of consensus on the measurement of TM. TM practices are mostly measured using self-constructed questionnaires from many different authors and references. There is no scale that measures TM practices overall and is broadly used in research (see Supplementary Table S3 for more information).

Fourth, concerning theories, the social-exchange theory (Homans, 1961; Blau, 2017) and the JD-R model (Schaufeli and Bakker, 2004) are the most used theories explaining relations among study variables (e.g., Memon et al., 2016; Kakkar et al., 2020). Both theories explain behaviors and relationships within the organizational context. The social exchange theory (SET) defends that employees will be inclined to stay in an organization if they perceive that this organization makes efforts toward them (Kumar, 2022). Therefore, if organizations invest in TM practices in order to develop employees and make them feel better, they will perceive this as important efforts toward them and will be engaged and willing to stay in the organization (Saks, 2006; Kloutsiniotis and Mihail, 2017; Memon et al., 2020). On the other hand, the JD-R theory explains that demands and resources act as antecedents to work engagement. In this case, TM practices act as job resources and are functional in developing personal resources, which act as positive antecedents of employee engagement (Kakkar et al., 2020; Memon et al., 2021).

**Research Question 2:** Which talent management activities are studied most recurrently in research regarding their relation with employee engagement and turnover intention?

Results showed that the five TM activities are frequently studied in relation to work or organizational engagement and intention to quit: selection and recruitment, training and development, rewards and recognition, performance management, and career development. Specifically, Rewards and Recognition is the most studied one. This can be explained by the fact that, traditionally, rewards have been the most popular motivator for people to stay in an organization and perform well (Kahn, 1990; Maslach et al., 2001); therefore, it seems only logical that researchers consider this practice when defining their hypotheses. However, literature in human resource management is growing, and activities other than rewards—such as performance management, career development, training and development, selection and recruitment—appear as promoters of employee engagement and intent to stay within an organization, broadening TM practices studied in research. Finally, Selection and Recruitment seems to be the one least studied. As proposed by Gladka et al. (2022), this activity is more usually related to the initial stages of the employee's life cycle, while employee retention may be more linked with middle and advanced stages of the employee life cycle; therefore, it seems logical that it is the one least studied.

**Research Question 3:** How is the relation between talent management practices, employee engagement, and turnover intention?

Concerning relations between study variables we could conclude that—broadly speaking—talent management practices have an influence on work and organizational engagement and turnover intention. Hence, they have a positive impact on work and organizational engagement, and a negative impact on turnover intention (e.g., van den Heuvel et al., 2017; Anjum and Din, 2022; Otoo, 2022). Although rewards and recognition resulted to be the most studied TM practice, some studies find a non-significant relationship with work or organizational engagement, or turnover intention (van der Merwe et al., 2020; Memon et al., 2021; Ramaprasad et al., 2021). This aligns with the growing relevance of the psychological contract for employees, which covers HRM topics beyond just rewards and money. In regard to mediating effects, it can be stated that the relationship between TM practices and turnover intention is mediated by work or organizational engagement (e.g., Kossyva et al., 2021; Rumawas, 2021; Sharma et al., 2022). Therefore, organizations should focus on developing and implementing TM practices that promote engagement of employees in order to reduce their employees' turnover intention. With regard to moderating variables, personal variables (work centrality and customer orientation) were found to moderate the relationship between study variables. This seems to be consistent with previous research, since these variables have a significant effect on engagement and turnover intention (Wu et al., 2017; Burawat, 2023).

**Research Question 4:** Which control variables are taken into account in the relationship between talent management practices, employee engagement, and turnover intention?

Gender, age, and organization tenure were the most used control variables, which is in line with previous results from literature (Bernerth and Aguinis, 2016). However, results show that gender does not correlate significantly with TM practices, work or organizational engagement, and turnover intention, while age and organizational tenure do (with exceptions) (see Supplementary Table S5 for more information). It is important to highlight that, in most of the studies, male participation was considerably higher than female participation (see Supplementary Table S1 for more information). Therefore, this might be the reason why gender has not been found to correlate significantly with study variables. Additionally, it has not been possible to explain the way in which control variables affect TM practices, engagement, and turnover intention, since age and tenure may not follow a normal distribution or include all kind of categories in the studies included.

Additionally, it is important to highlight that out of the 43 included studies, 24 have been published in the last 4 years. That is, once the COVID-19 pandemic hit the health, economic and organizational context worldwide. In other words, more studies addressing employee retention, engagement and talent management have been published in the past 4 years than in all the years prior to the pandemic. This shows a growing interest in understanding how organizations can promote employee engagement and retention in the post-pandemic world, characterized by a workforce looking for jobs that prioritize their wellbeing and happiness. Furthermore, it also highlights the collective effort both researchers and practitioners are making in order to understand how talent management needs to be done to retain their human capital in a constantly evolving organizational environment.

## Practical and theoretical implications

This study has grouped existing literature to confirm that talent management practices are functional in promoting work and organizational engagement, as well as in reducing turnover intention. Therefore, the main contribution of this study is confirming the relevance that talent management practices currently have for companies to adapt and survive to current changes in the environment, such as the talent shortage companies faced from 2021 to mid-2023 (Luna-Arocas and Danvila-del-Valle, 2022). Additionally, employee engagement is portrayed as a crucial factor for making employees want to stay in a company, that can be promoted through effective talent management practices adapted to different age groups and company tenures within the organization. Further, intention to stay of employees has always been related to organizational commitment (Humayra and Mahendra, 2019). This review defines employee engagement as a decisive variable—next to organizational commitment—in order to retain employees in a healthy and happy way.

These contributions have several implications for practitioners. First, they help them to understand the importance of putting effort in the design and implementation of TM practices in order to survive and succeed as a company, removing from their minds the thought that this is a waste of time. On the contrary, it has been demonstrated the positive effect of these practices in the retention and engagement of employees. Second, they become aware of the importance of all five TM practices considered in this review, since, despite some exceptions, they all affect turnover intention negatively, and employee engagement positively. Hence, it is important to focus on the whole TM process. Third, they realize they should not address everyone in the same way. Instead, they should focus on age and tenure differences when designing their TM practices and politics, addressing different groups in different ways. Finally, it provides practitioners with guidelines on how to address talent management issues in order to survive and succeed in the post-pandemic world.

The study also has implications for academics. First, this review offers a comprehensive analysis of which are the current trends in the talent management and organizational outcomes literature, offering an updated perspective on the most effective TM practices for retaining and engaging employees in a continuous evolving workplace environment. As it has been shown, TM literature has been addressed under different terms and perspectives, leading to a fragmentation of knowledge. This review aspires to unify all these names and perspectives to present a global understanding of the current state of TM literature, especially in relation with engagement and turnover intention. Second, this review has confirmed the lack of consensus on talent management names and definitions. However, a classification breakdown was made of all the names and definitions used to address these activities that can serve as a starting point for reaching a consensus on the definition and categorization of talent management. Third, this review can also serve as a starting point for focusing on organizational engagement as a mediating variable between employee outcomes and its antecedents since, in the studies where it is mentioned, it affects study variables in the expected direction.

## Limitations and future research

In spite of the relevant contributions of this research, it is also important to highlight its limitations. First, it should be considered that interesting articles may exist in other languages besides English or Spanish, given that the huge majority of articles studying this topic are published in Asia and Africa. Therefore, as detected in other systematic reviews (Kim et al., 2018; Cortés-Denia et al., 2023), it will be interesting for future research to work with reviewers who are fluent in other languages.

Second, this review has only included five TM practices, since they were the most relevant TM activities according to literature. The inclusion of more practices would have made this review less accurate and concise. However, more TM practices could be considered in the future for a wider review. For example, Oehley (2007) consider work-life balance, building and maintaining relationships, and providing meaningful work as important activities of TM. On the other hand, communication (Tash et al., 2016) and employee participation in decision-making (Takeuchi et al., 2009) may also be important TM activities. In the future, it will be also interesting to study the perceived organizational support for participating in talent management practices and activities, which has not been included in this review (Els et al., 2018; Kumar et al., 2018).

Third, this review has only analyzed the relationship between TM practices, engagement, and turnover intention in terms of how frequently they appear in research and the direction and significance of the proposed effects. Therefore, it may be interesting to conduct a meta-analysis in order to study the size of these relationships.

Finally, an interesting article (Ghosh et al., 2013) was excluded from the review since it did not fully meet the inclusion criteria, and its content and methodology differed too much from the other articles. However, it would be interesting to include it in future research, since it could yield compelling results.

## Future research agenda

As a result of the present review, below four topics are discussed since they seem highly relevant to further progress in TM, employee engagement, and turnover intention:

### Longitudinal multilevel design

In regard to study design, despite working with an organizational-level variable like talent management practices and individual-level variables like employee engagement and turnover intention, only five studies adopted a multilevel approach. Future research should focus on addressing a multilevel approach when studying these relationships, in order to get a closer and more accurate vision of the organizational context. Moreover, the majority of the studies used cross-sectional analysis, which has two limitations: (1) it increases the possibility of common method bias (CMB), since data is collected from a single source at a single point in time; and (2) causal relationships cannot be validated using this type of analysis. Both limitations can be solved by using longitudinal rather than cross-sectional analysis (Rindfleisch et al., 2008; González-Romá and Hernández, 2017).

### Organizational engagement

Organizational engagement is an antecedent of different positive organizational outcomes (Saks et al., 2022). However, according to the results of this review, studies examining it are scarce. Therefore, further research should focus on the concept of organizational engagement, since it shows itself as an important variable in retaining employees that has not been sufficiently studied so far (Saks et al., 2022).

### Universal talent management scale

As stated in RQ1, there is no accepted TM scale that measures the five TM activities considered in this review together. Instead, each author uses different scales to measure each of the studied practices (e.g., Juhdi et al., 2013; Shah and Beh, 2016; Dalal and Akdere, 2023). Therefore, a future line of research may be to develop a universal TM practices scale that meets reliability and validity criteria and integrates all main TM activities in one measurement scale, in order to provide more consistent results.

### Positive psychological interventions

Positive Organizational Psychology is gaining relevance as it helps to generate positive organizations, with high levels of employee engagement and retention intention (Salanova, 2021). The HERO model—developed by the WANT Research Team—encompasses the three elements of a positive organization, among which we can find: (1) positive organizational practices (TM practices), (2) healthy employees (e.g., engaged employees) and (3) healthy organizational outcomes (e.g., intention to stay). Following the HERO model, these three elements can be enhanced by positive psychological interventions (Salanova et al., 2012). Therefore, a future research line should be to develop positive psychological interventions in order to promote effective and healthy TM practices, engagement, and intention to stay.

## Conclusion

Studying the relation between talent management practices, employee engagement and turnover intention is crucial for practitioners to develop TM strategies for retaining engaged employees who, consequently, will be healthy, happy, and productive employees. This review has shed light to the numerous antecedents contributing to employee turnover, highlighting the relevant role that TM practices play in this phenomenon.

This results especially important in the current organizational environment since, although the Great Resignation has ended, organizations need to be prepared to face similar situations and crisis that may occur in the future. COVID-19 pandemic has made people to change their attitudes and expectations toward work, reconsidering their priorities in life (e.g., work-life balance). Retaining employees who have interiorized organization's values and have acquired valuable job knowledge is more efficient and cost-effective than constantly recruiting new employees. Also, organizations that retain this type of employees position themselves as excellent employers, attracting top talent in the current competitive organizational environment. Therefore, companies must adapt to these changes if they want to, not only retain employees, but also do it in a healthy and efficient way. A proactive approach to retaining and engaging employees serves as a preemptive measure, strengthening the organization against future challenges and crisis and contributing to its resilience.

In this context, it is essential to understand the underlying mechanisms that contribute to employee engagement and turnover intention. This review shows that, when addressed in an effective way, talent management practices can reduce the negative effects of turnover intention. A successful organization is an organization that adapts to the challenges of the environment. This review shows that investing efforts in TM activities is a proactive way to adapt to them.

In conclusion, this review presents solid conclusions that practitioners can use in order to engage and retain their employees. By investing in TM strategies, organizations will not only face a potential shortage of talent, but will also cultivate a healthy and engaged workforce, enabling them to emerge stronger and more resilient to the upcoming crisis.

## Data availability statement

The original contributions presented in the study are included in the article/Supplementary material, further inquiries can be directed to the corresponding author.

## Author contributions

LS: Conceptualization, Investigation, Methodology, Writing – original draft, Writing – review & editing. JP: Conceptualization, Methodology, Supervision, Validation, Writing – review & editing. MS: Conceptualization, Funding acquisition, Project administration, Supervision, Validation, Writing – review & editing.
