# Supplementary material for: Connecting the organizational incomes and outcomes: a systematic review of the relationship between talent management, employee engagement, and turnover intention
Source: Front Psychol. 2024 Jul 10;15:1439127. doi: 10.3389/fpsyg.2024.1439127 (PMC11278817; doi:10.3389/fpsyg.2024.1439127)
Supplement: Supplementary file 1 [file Table_1.docx]

**Supporting information**

**S1 Table. Methodological characteristics and summary of the included studies**

| **Reference and location** | | **N** | | **Sector** | | **Design** | | **Variables** | | | | | | | | | | **Theories** | |
| --- | --- | --- | --- | --- | --- | --- | --- | --- | --- | --- | --- | --- | --- | --- | --- | --- | --- | --- | --- |
|  |  |  |  |  |  |  |  | **Independent variable** | | **Dependent variable** | | **Mediating variable** | | **Moderating variable** | | **Control variables** | |  | |
| Albrecht & Marty (2020) (Australia) | | 623 (F: 43%) | | Multiple sectors | | Cross sectional | | Job autonomy  Skill utilization  Job feedback  Supervisor support  Development opportunities | | Turnover intention | | Work engagement  Affective commitment  Self-efficacy | | N/A | | N/A | | JD-R theory | |
| Alferaih et al. (2018) (Saudi Arabia) | | 521 (F:27,6%) | | Services (hotel) | | Cross sectional | | Extrinsic rewards  Role conflict  OC  Talent engagement  Job satisfaction | | Talent retention  Turnover intention | | N/A | | N/A | | N/A | | SET | |
| Alfes et al. (2013) (U.K.) | | 297 (F:52,2%) | | Services (business solutions) | | Cross sectional | | Perceived HRM practices | | Turnover intentions  OCB towards the organization | | Employee engagement | | POS  LMX | | Age  Gender  Full time/part time, Permanent/fixed term contract  Working hours  Job role | | SET | |
| Alhajaj & Ahmad (2023) (United Arab Emirates) | | 283 (F: 62,5%) | | Public sector | | Cross sectional | | Pay satisfaction  Empowerment  Participation and communication | | Turnover intention | | Work engagement | | Self-efficacy | | N/A | | JD-R theory | |
| Ang et al. (2013) (EE.UU) | | 251 (F:82%) | | Health (hospital) | | Cross sectional  multilevel | | Management perception of HPWS  Employee perception of HPWS | | Intention to leave  Affective commitment | | Work engagement  Job satisfaction | | N/A | | Permanent or not  Gender  Trust in supervisor | | SET | |
| Anjum and Din (2022) (Pakistan) | | N/A | | Education (university faculty members) | | Cross sectional | | HRM practices (career adaptability, performance appraisal) | | Turnover intention | | Work engagement | | Financial insecurity | | N/A | | SET | |
| Babakus et al. (2017) (Cyprus) | | 183 (F: 36%) | | Services (hotel) | | Longitudinal cross-lagged | | Stressors (challenge, hindrance)  HPWS (training, empowerment, rewards) | | Work engagement  Turnover intentions | | N/A | | Customer orientation | | N/A | | Transactional theory of stress  Job characteristics theory | |
| Bui & Chang (2018) (Vietnam) | | 470 (F: 67,7%) | | Public (public officials) | | Cross sectional | | Perceived soft talent management practices  Perceived hard talent management practices  Perceived person environment fit | | Turnover intention | | Employee engagement | | N/A | | N/A | | SET | |
| Dalal & Adkere (2021) (India) | | 992 (F: 21,6%) | | Industrial (manufacturing) | | Cross sectional | | TM Practices (talent acquisition, talent development, talent engagement, talent retention) | | Intent to stay  Job engagement  Affective commitment  Job satisfaction  Competency | | N/A | | Organizational culture | | N/A | | SET  Resource-Based View theory | |
| Ehrnrooth (2021) (Finland) | | 308 (F: 34%) | | Different industries | | Cross sectional | | Perceived transformational leadership  Perceived HPWS | | Employee attitudes:  Self-efficacy  Work engagement  Organizational identifications  Turnover intentions | | N/A | | Perceived HPWS | | Gender  Age  Tenure under the same supervisor  Industry | | AMO theory  SET  JD-R theory | |
| Fahmi et al. (2020) (Egypt) | | 208 (F: 42%) | | Education (higher education institutions) | | Cross sectional | | Talent management practices | | ITQ | | Work engagement | | N/A | | N/A | | N/A | |
| Gadi & Kee (2020) (Nigeria) | | 400 (F: 21,5%) | | Education (public universities) | | Cross sectional | | HRM practices (training and development, recruitment and selection, performance appraisal, rewards and recognition, career recognition)  Workplace bullying | | Turnover intention | | Work engagement | | N/A | | N/A | | SET  JD-R theory | |
| Herr et al. (2022) (Germany) | | 14182 (F: Cluster1=  26,17%;  Cluster2=  30%) | | Multiple sectors (industrial, services) | | Cross sectional | | Employee-friendliness of the company | | Mental Health  General health  Commitment  Work engagement  Turnover intention  Job satisfaction | | N/A | | N/A | | Age  Gender  White-or-blue-collar  Company size  Industry | | N/A | |
| Islam et al. (2023)  (Bangladesh) | | 343 (F:10%) | | Financial (banks) | | Cross sectional | | HR practices (empowerment, information sharing, rewards and training) | | Turnover intention | | Work engagement | | Functional competence | | N/A | | JD-R | |
| Juhdi et al. (2013) (Malaysia) | | 457 (F: 55%) | | Multiple sectors (finance, education) | | Cross sectional | | HR practices (career management, person-job fit, pay satisfaction, performance appraisal, job control) | | Turnover intention | | Organizational commitment  Organizational engagement | | N/A | | N/A | | N/A | |
| Kakkar et al. (2020) (India) | | 322 (F: 12,4%) | | Multiple sectors | | Cross sectional | | Perceptions of Performance Management System effectiveness | | Job satisfaction  Turnover intention | | Work engagement | | N/A | | Age  Gender  Work experience | | SET  JD-R | |
| Katou (2017) (Greece) | | 996 (F: 39,5%) | | Multiple sectors (manufacturing, service, trade) | | Cross sectional  multilevel | | Business strategies | | Performance | | HRM content (resourcing, development, rewards, relations)  HRM process  HRM as experienced (resourcing, development, rewards, relations)  Employee attitudes (job satisfaction, motivation, OC)  Employee behavior (work engagement, OCB, co-operation, ITQ) | | N/A | | Sector  Organizational size | | Attribution theory | |
| Kloutsiniotis & Mihail (2017) (Greece) | | 296 (F: 50%) | | Health (health care institutions) | | Cross sectional | | HPWS | | Intention to leave  Affective commitment | | Work engagement  Job Satisfaction | | N/A | | Age  Gender  Education | | SET  AMO framework | |
| Kossyva et al. (2021) (Multiple countries) | | 499 (F:40,3%) | | Multiple sectors (IT, financial, trade) | | Cross sectional | | HPWS | | Turnover intention | | Change management practices  Work engagement | | N/A | | N/A | | AMO framework | |
| Kossyva et al. (2024)  (Multiple countries) | | 168 (F:44,6%) | | Multiple sectors | | Cross sectional | | HRM Practices | | Turnover intention | | Change management practices  Knowledge management practices  Employee engagement | | N/A | | N/A | | Conservation of resources theory  JD-R theory | |
| Lee et al. (2019) (Malaysia) | | 500 (F: 56,6%) | | Services | | Cross sectional multilevel | | Transactional leadership  Transformational leadership | | Turnover intention | | Supervisory coaching  Performance feedback  Work engagement | | N/A | | Marital status | | JD-R theory | |
| Marescaux et al. (2013) (Belgium) | | 5748 (F: 44%) | | Multiple sectors (administration, trade, ICT) | | Cross sectional | | HRM practices (career development, training, direct employee participation, developmental appraisal, mentoring) | | Turnover intention | | Basic need satisfaction  Work engagement  Affective organizational commitment | | N/A | | Gender  Age  Education  Position  Organizational tenure | | Self-determination theory | |
| Memon et al. (2016) (Malaysia) | | 409 (F: 43,5%) | | Industrial (oil and gas manufacturing) | | Cross sectional | | Training satisfaction | | Turnover intention | | Work engagement | | N/A | | N/A | | SET  JD-R theory | |
| Memon et al. (2017) (Malaysia) | | 415 (F:44%) | | Industrial (oil and gas manufacturing) | | Cross sectional | | Pay satisfaction | | Turnover intention | | Work engagement | | N/A | | N/A | | SET  Equity theory | |
| Memon et al. (2020) (Malaysia) | | 295 (F: 36%) | | Industrial (oil and gas manufacturing) | | Longitudinal cross-lagged | | Performance appraisal satisfaction | | Turnover intention | | Work engagement | | N/A | | N/A | | SET | |
| Memon et al. (2021) (Malaysia) | | 442 (F:43,2%) | | Multiple sectors | | Cross sectional | | Training satisfaction  Performance appraisal satisfaction  Pay satisfaction | | Turnover intention | | Work engagement | | N/A | | N/A | | JD-R theory | |
| Oliveira et al. (2015) (Brazil) | | 189 (F:38%) | | Non-profit organization | | Cross sectional | | Employees evaluation of HPWS  Leader member exchange quality | | Turnover intention | | Work engagement | | N/A | | Gender  Age  Organizational tenure | | LMX | |
| Oliveira & Rocha (2017) (Brazil) | | 303 (F:45%) | | Multiple sectors (industrial, services) | | Cross sectional | | Core-self evaluations  High Performance HRM practices  LMX quality | | Turnover intention | | Work engagement | | N/A | | Gender  Age  Management position  Organizational tenure  Company size  Company type | | LMX  AMO framework | |
| Otoo (2022) (Ghana) | | 900 (F: 43,3%) | | Health (health care institutions) | | Cross sectional | | Human Resource Development | | Employee turnover intentions | | Employee engagement | | N/A | | N/A | | SET | |
| Ramaprasad et al. (2021) (India) | | 752 (F: 35,3%) | | Technology (IT) | | Cross sectional | | HPWS | | Turnover intention | | Work engagement | | N/A | | Age  Gender  Organizational tenure No. of job offers | | SET  AMO framework | |
| Rezwan & Takahashi (2021) (Bangladesh) | | 175 (F:24%) | | Financial (banks) | | Cross sectional | | Proactive personality | | Retention intention | | Work engagement | | High Performance Human  Resource Practices | | Gender  Age  Education  Current job level  Tenure  Total experience | | N/A | |
| Rumawas (2021) (Indonesia) | | 182 (F: 53,8%) | | Financial (banks) | | Cross sectional | | TM practices | | Turnover intention | | Employee engagement  POS | | N/A | | N/A | | SET | |
| Saks (2006) (Canada) | | 102 (F: 60%) | | Multiple sectors | | Cross sectional | | Job characteristics  Perceived supervisor support  Perceived organizational support  Rewards and recognition  Procedural justice  Distributive justice | | Job satisfaction  Organizational commitment  ITQ  OCB | | Employee engagement | | N/A | | N/A | | SET | |
| Shah & Beh (2016) (Malaysia) | | 401 (n/a) | | Services (hotel) | | Cross sectional | | Motivation enhancing practices | | Turnover intention | | Employee engagement | | N/A | | N/A | | SET | |
| Sharma et al. (2022) (India) | | 327 (F: 23%) | | Multiple sectors (manufacturing, services) | | Cross sectional | | Performance management system efficiency | | Turnover intention | | Psychological contract fulfillment  Work engagement | | N/A | | POS | | Equity theory  SET | |
| Sheehan et al. (2019) (Australia) | | 1039 (F: 92%) | | Health | | Cross sectional | | Career development  Job content  Social atmosphere  Financial rewards  Work-life balance | | Intention to leave | | Work engagement | | N/A | | Setting of employment  Age  Organizational tenure  Hours worked per week | | SET  Cognitive dissonance theory | |
| Sousa et al. (2021) (Portugal) | | 802 (F: 55,6%) | | Multiple sectors | | Cross sectional | | Age-diversity practices | | Turnover intention | | Work engagement  Affective commitment | | Work centrality | | Age | | Signaling theory  SET | |
| van den Heuvel (2017) (The Netherlands) | | 669 (F: 20%) | | IT (Technology) | | Cross sectional | | Change information | | Turnover intention | | Work engagement  Psychological contract fulfillment  Trust  Attitude towards change | | N/A | | N/A | | SET | |
| van der Merwe et al. (2020) (South Africa) | | 102 (F: 25%) | | Financial | | Cross sectional | | Demands (surface and deep acting, time wasted)  Resources (advanced growth opportunities, pay satisfaction, Perceived supervisor support) | | ITQ | | Work engagement | | Psychological capital | | N/A | | SET | |
| Wen et al. (2022) (China) | | 1219 (F: 36,8%) | | Health (pharmaceutical) | | Cross sectional | | Pay satisfaction  Satisfaction with supervisor  Satisfaction with coworkers  Satisfaction with promotion  Satisfaction with work itself | | Turnover intention | | Employee engagement | | Position level | | N/A | | N/A | |
| Winarno et al. (2022) (Indonesia) | | 377 (F: n/a) | | Services (transportation) | | Cross sectional | | Human resource practices | | Turnover intention | | POS  Work engagement | | N/A | | N/A | | SET | |
| Yusliza et al. (2021) (Malaysia) | | 202 (n/a) | | Industrial (manufacturing) | | Cross sectional | | Green Human Resource Management | | Turnover intention  Job performance | | Employee engagement | | POS | | N/A | | SET | |
| Zhong et al. (2016) (China) | | 865 (F: 53,22%) | | Multiple sectors | | Cross sectional multilevel | | High-Performance Human Resource Practices | | In role performance  OCB  Intention to quit | | Work engagement  POS | | Collectivism  Power distance | | Industry charachteristics  Company type  Company size and tenure  Supervisor sex  Age, sex | | SET | |

Abbreviations: F, female; ITQ, intention to quit; OCB, organizational citizenship behavior; N/A, not applicable/ not available; SET, social-exchange theory; HPWS. high-performance work system; OC, organizational commitment; POS, perceived organizational support; LMX, leader-member exchange; HRM, human resource practices

**S2 Table. Nomenclature and categorization of TM practices**

| **Reference** | **Concept/construct** | **Definition** |
| --- | --- | --- |
| Albrecht & Mary (2020) | Job Resources | No definition provided |
| Alferaih et al. (2018) | A practice | N/A |
| Alfes et al. (2013) | Human Resource Management Practices | No definition provided |
| Alhajaj & Ahmad (2023) | Human Resource Management Practices | No definition provided |
| Ang et al. (2013) | High Performance Work Systems | No definition provided |
| Anjum and Din (2022) | A practice | N/A |
| Babakus et al. (2017) | High Performance Work Practices | No definition provided |
| Bui & Chang (2018) | Talent Management | The systematic attraction, identification, development, retention and deployment of those individuals with high potential which are of special value to an organization |
| Dalal & Akdere (2021) | Talent Management Practices | “TM has been viewed from different perspectives in the literature (Gallardo-Gallardo et al., 2015; Hambrick &  Mason, 1984; Huselid et al., 1997; Miller et al., 1998; Schuler, 1989; Swailes, 2016; Wright & McMahan, 1992). For example, Blass (2007) related TM only to the people who are considered to be talented in an organization, whereas Jackson and Schuler (1990) related TM to the processes that ensure there are an adequate number of  employees for jobs within an organization (as cited in Sita & Pinapati, 2013). Practices of TM are similar to various HRD functions, including organization development and performance management (Lewis & Heckman, 2006). Cappelli (2008) explained TM in the simplistic way, “At its heart,  TM is simply a matter of anticipating the need for human capital and then setting out a plan to meet it” (p. 1).” (p.4) |
| Ehrnrooth et al. (2021) | High Performance Work System | A bundle of discrete but interrelated HRM policies and practices, rather than isolated practices, to select, develop, retain and motivate employees to reach superior indicators of organizational performance |
| Fahmi et al. (2020) | Talent Management Practices | A unique process that only focuses on those persons who have the capacity to provide competitive advantage by managing those people effectively and efficiently |
| Gadi & Kee (2020) | Human Resource Management Practices | HR policies that can influence employee’s contribution to organizational outcomes |
| Herr et al. (2022) | Employee-friendly company | N/A |
| Islam et al. (2023) | High-Involvement Work Practices | “A symbiotic collection of practices concerning power, information sharing, rewards and knowledge that empower employees” (p.1635) |
| Juhdi et al. (2013) | Human Resource Management practices | The ways that organizations use to mold employee behavior, perception and attitude |
| Kakkar et al. (2020) | A practice | N/A |
| Katou (2017) | Human Resource Management | A set of HR practices through which organizations improve acquisition, development, retention and utilization of their human capital to achieve organization goals |
| Kloutsiniotis & Mihail (2017) | High Performance Work Systems | Specific combination of HR practices, work structures and processes that promote and maximizes employee commitment, abilities, knowledge and flexibility |
| Kossyva et al. (2021) | High-performance work systems | Collection of HR practices which form an integrated approach designed to motivate employees to perform at their highest potential |
| Kossyva et al. (2024) | Human Resource Management | “A formal process within an organization that aims to attract, retain and develop human talents to achieve organizational sustainability and gain competitive advantages” (p. 412) |
| Lee et al. (2019) | Human Resource Development | The field of study and practices in charge of enhancing a long-term, work–related learning capacity at the individual, group and organizational level of organizations |
| Marescaux et al. (2013) | Human Resource Management | All activities related to the management of people in firms |
| Memon et al. (2016) | A practice | N/A |
| Memon et al. (2017) | A practice | N/A |
| Memon et al. (2020) | A practice | N/A |
| Memon et al. (2021) | Human Resource Management Practices | A collection of individual but internally consistent practices aimed to strength an organization’s human capital in consonance with its business objectives |
| Oliveira et al. (2015) | High Performance Work Systems | A bunch of HRM practices arranged in order to promote knowledge, abilities and skills of employees, enhance motivation and foster an environment favorable to their contributions, generating convenient conditions for the development of resources and capabilities that create value for the organization |
| Oliveira & Rocha (2017) | High Performance Work System | HR practices and policies interrelated which contribute towards the organization’s strategic and business goals |
| Otoo (2022) | Human Resource Development | A set of coordinated actions undertaken over a predetermined period of time intended  to generate behavioral change. A collection of organized and unstructured performance-based learning activities that increases an organization and individual  capacity to successfully manage change. |
| Ramaprasad et al. (2021) | High Performance Work Systems | A configuration of coherent and synergetic HRM practices (ability-enhancing, motivation-enhancing and opportunity-enhancing). HRM refers to the choices organizations made from among the practices available for managing manpower |
| Rezwan & Takahashi (2021) | High Performance Human Resource Practices | A group of interrelated human resource management practices oriented towards enhancing organizational flexibility and participatory aspects for better performance by improving employee’s abilities and motivation. |
| Rumawas (2021) | Talent Management Practices | A set of long-term organizational strategies that enhance human capital to the organization’s competitive advantage, in addition to a portfolio of integrated human resource activities which result in the placement of the right people with the right skills and abilities in the right job in the right place and at the right cost. |
| Saks (2006) | Antecedents of engagement | N/A |
| Shah & Beh (2016) | Human Resource Management Practices | “A professional approach to manage people in the organization by the using the different set of professional practices which are integrated to ensure organizational success. The directing, organizing, planning, controlling, integrating, compensating and developing of HR so that the organization achieves its goal and objectives. HR practices play a vital role in retention of customers.” (p.824) |
| Sharma et al. (2022) | A practice | N/A |
| Sheehan et al. (2019) | Psychological Contract | N/A |
| Sousa et al. (2021) | Age-diversity practices | Perception of workers that organizational practices are age-inclusive and sensitive, accompanied by age-related changes in workers’ needs and goals. |
| van den Heuvel (2017) | Psychological Contract | N/A |
| van der Merwe et al. (2020) | Job Resources | The physical, psychological, social or organizational aspects of work that can reduce the negative impact on health of job demands, help to achieve work goals and stimulate personal growth and development |
| Wen et al. (2022) | A practice | N/A |
| Winarno et al. (2022) | Human Resource Management Practices | No definition provided |
| Yusliza et al. (2021) | Green Human Resource Management | Adjustment of HRM practices with environmental goals of organization |
| Zhong et al. (2016) | High Performance Human Resource Practices | A combination of unique but interconnected HR practices that serve as signals about an organization’s investment on employees |

**S3 Table. Instruments and scales**

| **Reference** | **TM practices scale** | **Engagement scale** | **Turnover intention scale** |
| --- | --- | --- | --- |
| Albrecht & Marty (2020) | Bakker, Demerouti, and Euwema (2005); Xanthopoulou et al. (2007)  ɑ=.86-.93 | UWES-9 (Schaufeli et al., 2006)  ɑ=.81-.84 | Seashore et al. (1982); Vigoda-Gadot and Kapun (2005)  ɑ=.85 |
| Alferaih et al. (2018) | Self-constructed  ɑ=.774 | Self-constructed  ɑ=.869 | Self-constructed  ɑ=.856 |
| Alfes et al. (2013) | Gould-Williamsand Davies (2005)  ɑ=.77 | Rich et al. (2010)  ɑ=.88 | Boroff and Lewin (1997)  ɑ=.95 |
| Alhajaj & Ahmad (2023) | Heneman and Schwab (1985)  ɑ = .929 | UWES-9 (Schaufeli et al., 2006)  ɑ = .94 | Jung & Noon (2013)  ɑ = .926 |
| Ang et al. (2013) | Self-constructed from Zacharatos  et al. (2005)  ɑ=.821-.89 | Demerouti et al. (2001)  ɑ=.809-.903 | Mobley et al. (1978)  ɑ=.889-.941 |
| Anjum and Din (2022) | Chang et al. (2007); Boswell and Boudreau (2000)  ɑ=.775 | Bakker and Albrecht (2018); Schaufeli et al. (2002)  ɑ=.710 | Lum et al. (1998); Wayne, Shore, and Liden (1997)  ɑ=.857 |
| Babakus et al. (2017) | Boshoff and Allen (2000)  ɑ=.78-.92 | Oldenburg Burnout Inventory  ɑ=.71 | Singhet al. (1996)  ɑ=.91 |
| Bui & Chang (2018) | Boon et al., (2011); Sadangharn (2010)  ɑ=.865 | Saks (2006); Rich et al. (2010)  ɑ=.832 | Sadangharn (2010)  ɑ=.847 |
| Dalal & Akdere (2021) | Self-constructed from different authors (Pattnaik & Misra. (2014); Holton III. (1990); Odunlade. (2012); Dipboye & De Pontbriand. (1981); Yi. (2009); Zaniboni et al. (2011); Mitchel et al. (2001); Eisenberger et al. (1986); Brook et (2007); Price & Mueller. (1986)  ɑ=.84-.88 | Job engagement survey (Rich et al., 2010)  ɑ=.85 | Intento to stay scale (Hoisch, 2001)  ɑ= .74 |
| Ehrnrooth et al. (2021) | Sun, Aryee, and Law's (2007)  ɑ=n/a | UWES-9 (Schaufeli et al., 2006)  ɑ=.88 | Rusbult et al. (1988)  ɑ=.83 |
| Fahmi et al. (2020) | Human capital Index (Human Capital Institute, 2008)  ɑ=.89 | UWES-9 (Schaufeli et al., 2006)  ɑ=.91 | Intention to quit scale (Arnold & Feldman, 1982)  ɑ=.87 |
| Gadi & Kee (2020) | Langford (2009)  ɑ=.89 | UWES-9 (Schaufeli et al., 2006)  ɑ=.92 | Bothma and Roodt (2013)  ɑ=.9 |
| Herr et al. (2022) | Self-constructed  ɑ=n/a | UWES-9 (Schaufeli et al., 2006)  ɑ=.91 | Self-constructed  ɑ=n/a |
| Islam et al. (2023) | Riordan et al. (2005)  Rewards ɑ = .866  Training ɑ= .833 | UWES-3 (Schaufeli et al., 2017)  ɑ = .787 | Meyer et al. (1993)  ɑ = .922 |
| Juhdi et al. (2013) | Different authors: Dulebohn and Ferris (1999); Sturges, Guest and Davey (2000); Cable and Judge (1996); Abdel Halim (1981); Smith (1976)  ɑ=.813-.879 | Saks (2006)  ɑ=.837 | Cammann, Fichman, Jenkins and Klesh (1979)  ɑ=.898 |
| Kakkar et al. (2020) | Sharma et al. (2016)  ɑ= .94 | UWES-9 (Schaufeli et al., 2006)  ɑ=.85 | MOAQ-TOI (Bowling and Hammond,  2008)  ɑ=.79 |
| Katou (2017) | Kinnieet al. (2005)  ɑ=.935 | UWES-9 (Schaufeli et al., 2006)  ɑ=.855 | Firthet al. (2004)  ɑ=.855 |
| Kloutsiniotis & Mihail (2017) | Self-constructed from different authors: Anget al. (2013); Delery and Doty (1996); Zacharatoset al. (2005)  ɑ= .915 | Oldenburg Burnout Inventory (OLBI) (Demerouti et al., 2010)  ɑ=.7 | Ang et al. (2013)  ɑ=.887 |
| Kossyva et al. (2021) | Bhatti et al. (2020); Edgar et al. (2021); Tian et al. (2016)  ɑ=.901 | Shuck et al. (2017)  ɑ=.89 | Meyer et al. (1993)  ɑ=.894 |
| Kossyva et al. (2024) | Bhatti et al. (2020); Katou and Budhwar (2010); Tian et al. (2016)  ɑ = .85 | Shuck et al. (2017); Shuck et al. (2014)  ɑ = .88 | Cammann et al. (1983)  ɑ = N/A |
| Lee et al. (2019) | Bakker et al., (2013)  ɑ=.85 | UWES-9 (Schaufeli et al., 2006)  ɑ=.9188 | O’Driscoll and Beehr (1994)  ɑ=.90 |
| Marescaux et al. (2013) | Self-constructed  ɑ=n/a | UWES-9 (Schaufeli et al., 2006)  ɑ=.94 | Jiang and Klein’s(2002)  ɑ=.93 |
| Memon et al. (2016) | Schmidt (2007)  ɑ= .91 | UWES-9 (Schaufeli et al., 2006)  ɑ=.89 | Jung and Yoon (2013)  ɑ=.92 |
| Memon et al. (2017) | Scarpello and Carraher (2008)  ɑ= .91 | UWES-9 (Schaufeli et al., 2006)  ɑ=.90 | Jung and Yoon (2013)  ɑ=.86 |
| Memon et al. (2020) | Miller (2001)  ɑ= .71 | UWES-9 (Schaufeli et al., 2006)  ɑ= .92 | Jung and Yoon (2013)  ɑ=.86 |
| Memon et al. (2021) | Schmidt (2006, 2007); Miller (2001); Heneman & Schwab (1985)  ɑ= .71- .90 | UWES-9 (Schaufeli et al., 2006)  ɑ= .90 | Jung and Yoon (2013)  ɑ=.86 |
| Oliveira et al. (2015) | Sun et al. (2007); Deen Hartog et al. (2012); Jensen et al. (2011)  ɑ= .94 | UWES-17 (Schaufeli et al., 2006)  ɑ= .95 | Jensen et al. (2011)  ɑ= .82 |
| de Oliveira & Rocha (2017) | Jiang et al. (2012)  ɑ= .945 | UWES-9 (Schaufeli et al., 2006)  ɑ= .959 | Jensen et al. (2013)  ɑ= .894 |
| Otoo (2022) | PIED scale (Lee and Bruvold, 2003)  ɑ= .94 | Different authors: Rich et al. (2010); Shuck et al. (2014); Macey & Schneider (2008)  ɑ= .88 | Cho et al. (2002)  ɑ= .82 |
| Ramaprasad et al. (2021) | Self-constructed  ɑ= .78- .92 | UWES-9 (Schaufeli et al., 2006)  ɑ= .86- .9 | Shore & Martin (1989)  ɑ= .839 |
| Rezwan & Takahashi (2021) | Kehoe & Wright (2013)  ɑ=n/a | UWES-9 (Schaufeli et al., 2006)  ɑ= .9 | Kehoe & Wright (2013)  ɑ= .813 |
| Rumawas (2021) | Capital index of human Capital Institute Vurv Technology (2008)  ɑ= .958 | UWES-9 (Schaufeli et al., 2006)  ɑ= .872 | Madden et al. (1992)  ɑ= .933 |
| Saks (2006) | Saks (2006)  ɑ= .80 | Saks (2006)  ɑ= .82- .90 | Colarelli’s (1984)  ɑ=.82 |
| Shah & Beh (2016) | Different authors: Tremblay et al. (1997); Gardner et al. (2011); Lawrence and Jordan (2009); Spector and Jex (1998); Akhtar et al. (2008); Isaksson et al. (1998)  ɑ= .82 | Saks (2006)  ɑ=.79- .83 | Farh et al. (1998)  ɑ=.88 |
| Sharma et al. (2022) | Sharma et al. (2016); Colquitt's (2001) ɑ= .86- .89 | UWES-9 (Schaufeli et al., 2006)  ɑ=.89 | MOAQ (Camman et al., 1983)  ɑ=.77 |
| Sheehan et al. (2019) | De Voset al.(2003)  ɑ= .9 - 94 | UWES-9 (Schaufeli et al., 2006)  ɑ=-91 | Meyer et al. (1993)  ɑ = .8 |
| Sousa et al. (2021) | Sousa et al. (2019)  ɑ=.87 | UWES-9 (Schaufeli et al., 2006)  ɑ = .94 | Self-constructed  ɑ=.84 |
| van den Heuvel (2017) | Freese et al.(2008)  ɑ=n/a | UWES-9 (Schaufeli et al., 2006)  ɑ = .89 | Freese (2007)  ɑ=.81 |
| van der Merwe et al. (2020) | Different authors: Heneman & Schwab (1985); Rothmann & Jordaan (2006)  ɑ=.83-.93 | UWES-9 (Schaufeli et al., 2006)  ɑ=.90 | Oehley, 2007  ɑ=.87 |
| Wen et al. (2022) | Self-constructed  ɑ=.815-.82 | Self-constructed  ɑ=.739 | Self-constructed  ɑ=.802 |
| Winarno et al. (2022) | Tessema and Soeters (2006)  ɑ=.98 | Schaufeli et al. (2002)  ɑ=.956 | Prasetio et al. (2020)  ɑ=.925 |
| Yusliza et al. (2021) | Self-constructed from different authors: Williams and Anderson (1991); Thatcher et al. (2002); Jabbour (2011)  ɑ=.887 | Saks (2006)  ɑ=.95 | Eisenberger et al. (1986)  ɑ = .973 |
| Zhong et al. (2016) | Sun et al. (2007)  ɑ=.83 | Rich et al. (2010)  ɑ=.92 | Wayne et al. (1997)  ɑ=n/a |

**S4 Table. Summary of the relation between talent management, work engagement and turnover intention**

| **Reference** | **TM → TI** | **TM → EE** | **Mediation** | **Moderation** |
| --- | --- | --- | --- | --- |
| Albrecht & Marty (2020) | N/A | SIGNIFICANT POSITIVE EFFECT | Total mediation* | N/A |
| Alferaih et al. (2018) | SIGNIFICANT NEGATIVE EFFECT | N/A | N/A | N/A |
| Alfes et al. (2013) | SIGNIFICANT NEGATIVE EFFECT | SIGNIFICANT POSITIVE EFFECT | HRMP→EE→TI: PARTIAL MEDIATION | POS moderates relation between WE and TI  LMX doesn’t moderate relation between WE and TI |
| Alhajaj & Ahmad (2023) | SIGNIFICANT NEGATIVE EFFECT | SIGNIFICANT POSITIVE EFFECT | Pay satisfaction 🡪 WE 🡪 TI: PARTIAL MEDIATION | Self-efficacy does not moderate the relationship between work engagement and turnover intention |
| Ang et al. (2013) | N/A | Different results for different samples  Significant positive effect (except nursing group) | Different results for different samples  Mediation (except administration employee group) | N/A |
| Anjum and Din (2022) | SIGNIFICANT NEGATIVE EFFECT | SIGNIFICANT POSITIVE EFFECT | HRMP→WE→TI: MEDIATION | Financial uncertainty moderates relation between WE and TI |
| Babakus et al. (2017) | SIGNIFICANT NEGATIVE EFFECT (Training no significant effect) | NO SIGNIFICANT EFFECT | N/A | Customer orientation moderates all the relationships |
| Bui & Chang (2018) | Soft TM practices: NO SIGNIFICANT EFFECT  Hard TM practices: SIGNIFICANT NEGATIVE EFFECT | Soft TM practices: NO SIGNIFICANT EFFECT  Hard TM practices: SIGNIFICANT POSITIVE EFFECT | Soft TMP→EE→TI: NO MEDIATION  Hard TMP→EE→TI: TOTAL MEDIATION | N/A |
| Dalal & Akdere (2021) | SIGNIFICANT NEGATIVE EFFECT | SIGNIFICANT POSITIVE EFFECT | N/A | Organizational culture doesn’t moderate relations |
| Ehrnrooth et al. (2021) | SIGNIFICANT NEGATIVE EFFECT | SIGNIFICANT POSITIVE EFFECT | N/A | HPWS doesn’t mediate relations between transformational leadership and WE nor TI |
| Fahmi et al. (2020) | SIGNIFICANT NEGATIVE EFFECT | SIGNIFICANT POSITIVE EFFECT | TMP→WE→TI: PARTIAL MEDIATION | N/A |
| Gadi & Kee (2020) | N/A | SIGNIFICANT POSITIVE EFFECT | HRMP→WE→TI: MEDIATION | N/A |
| Herr et al. (2022) | SIGNIFICANT NEGATIVE EFFECT | SIGNIFICANT POSITIVE EFFECT | N/A | N/A |
| Islam et al. (2023) | SIGNIFICANT NEGATIVE EFFECT | NOT SIGNIFICANT | NO MEDIATION | Functional competence negatively moderates the relationship between rewards and work engagement  Functional competence does not moderate the relationship between training and work engagement |
| Juhdi et al. (2013) | SIGNIFICANT NEGATIVE EFFECT  (Career management no significant effect) | SIGNIFICANT POSITIVE EFFECT  (Career management strongest predictor) | TM practices→OE→TI: Partial mediation | N/A |
| Kakkar et al. (2020) | SIGNIFICANT NEGATIVE EFFECT | SIGNIFICANT POSITIVE EFFECT | PMS→WE→TI: PARTIAL MEDIATION | N/A |
| Katou (2017) | N/A | N/A | Total mediation | N/A |
| Kloutsiniotis & Mihail (2017) | SIGNIFICANT NEGATIVE EFFECT | SIGNIFICANT POSITIVE EFFECT | HPWS→WE→TI: PARTIAL MEDIATION | N/A |
| Kossyva et al. (2021) | SIGNIFICANT NEGATIVE EFFECT | SIGNIFICANT POSITIVE EFFECT | HPWS→WE→TI: MEDIATION | N/A |
| Kossyva et al. (2024) | N/A | SIGNIFICANT POSITIVE EFFECT | HRM 🡪 EE 🡪 TI: MEDIATION | N/A |
| Lee et al. (2019) | N/A | SIGNIFICANT POSITIVE EFFECT | PF→WE→TI: MEDIATION | N/A |
| Marescaux et al. (2013) | SIGNIFICANT NEGATIVE EFFECT | SIGNIFICANT POSITIVE EFFECT | Mediation* | N/A |
| Memon et al. (2016) | SIGNIFICANT NEGATIVE EFFECT | SIGNIFICANT POSITIVE EFFECT | TS→WE→TI: MEDIATION | N/A |
| Memon et al. (2017) | N/A | SIGNIFICANT POSITIVE EFFECT | PS→WE→TI: MEDIATION | N/A |
| Memon et al. (2020) | N/A | SIGNIFICANT POSITIVE EFFECT | PAS→WE→TI: MEDIATION | N/A |
| Memon et al. (2021) | N/A | SIGNIFICANT POSITIVE EFFECT  (Rewards no significant effect) | TS→WE→TI: MEDIATION  PAS→WE→TI: MEDIATION  PS→WE→TI: NO MEDIATION | N/A |
| Oliveira et al. (2015) | N/A | SIGNIFICANT POSITIVE EFFECT | Mediation* | N/A |
| Oliveira & Rocha (2017) | N/A | SIGNIFICANT POSITIVE EFFECT | Mediation* | N/A |
| Otoo (2022) | SIGNIFICANT NEGATIVE EFFECT | SIGNIFICANT POSITIVE EFFECT | HRD→EE→TI: MEDIATION | N/A |
| Ramaprasad et al. (2021) | SIGNIFICANT NEGATIVE EFFECT  (Rewards no significant effect) | SIGNIFICANT POSITIVE EFFECT | HPWS→WE→TI: TOTAL MEDIATION | N/A |
| Rezwan & Takahashi (2021) | N/A | N/A | Proactive personality→WE→TI: MEDIATION | HRHRP doesn’t moderate relation between proactive personality and EE nor ITQ |
| Rumawas (2021) | SIGNIFICANT NEGATIVE EFFECT | SIGNIFICANT POSITIVE EFFECT | TMP→EE→TI: MEDIATION | N/A |
| Saks (2006) | N/A | NO SIGNIFICANT EFFECT | RR→EE→TI: PARTIAL MEDIATION | N/A |
| Shah & Beh (2016) | SIGNIFICANT NEGATIVE EFFECT | N/A | MOT→WE→TI: NO MEDIATION  MOT→OE→TI: PARTIAL MEDIATION | N/A |
| Sharma et al. (2022) | SIGNIFICANT NEGATIVE EFFECT | SIGNIFICANT POSITIVE EFFECT | PMSE →WE →TI: MEDIATION | N/A |
| Sheehan et al. (2019) | NO SIGNIFICANT EFFECT | NO SIGNIFICANT EFFECT | NO MEDIATION | N/A |
| Sousa et al. (2021) | NO SIGNIFICANT EFFECT | SIGNIFICANT POSITIVE EFFECT | Age-diversity practices →WE→TI: MEDIATION | Work centrality moderates relation between Age-diversity practices and EE |
| van den Heuvel (2017) | SIGNIFICANT NEGATIVE EFFECT | SIGNIFICANT POSITIVE EFFECT | Mediation* | N/A |
| van der Merwe et al. (2020) | SIGNIFICANT NEGATIVE EFFECT  (Rewards no significant effect) | SIGNIFICANT POSITIVE EFFECT  (Rewards no significant effect) | CD→ WE→TI: PARTIAL MEDIATION  PS→WE→TI: NO MEDIATION | N/A |
| Wen et al. (2022) | SIGNIFICANT NEGATIVE EFFECT | SIGNIFICANT POSITIVE EFFECT | SP→EE→TI: PARTIAL MEDIATION  PS→EE→TI: NO MEDIATION | Position level doesn’t moderate relations |
| Winarno et al. (2022) | NO SIGNIFICANT EFFECT | SIGNIFICANT POSITIVE EFFECT | HRP→WE→TI: MEDIATION | N/A |
| Yusliza et al. (2021) | N/A | SIGNIFICANT POSITIVE EFFECT | GHRM→EE→TI: MEDIATION | POS moderates relation between EE and TI |
| Zhong et al. (2016) | N/A | SIGNIFICANT POSITIVE EFFECT | Mediation | N/A |

Abbreviations: TI, Turnover intention

**S5 Table. Summary of the relation between control variables and TM practices, employee engagement, and turnover intention**

| **Reference** | **Justification** | **Correlations with study variables** | | |
| --- | --- | --- | --- | --- |
|  |  | **Control variable: Gender** | **Control variable: Age** | **Control variable: Organization tenure** |
| Alfes et al. (2013) | Relation with perception of HR practices (Kinnie et al., 2005) | Not significant | Not significant | - |
| Ang et al. (2013) | N/A | Not significant | - | - |
| Ehrnrooth et al. (2021) | N/A | Not significant | -.18** Turnover intention | - |
| Herr et al. (2022) | N/A | N/A | N/A | N/A |
| Kakkar et al. (2020) | N/A | N/A | N/A | - |
| Kloutsiniotis & Mihail (2017) | N/A | Not significant | Not significant | - |
| Marescaux et al. (2013) | There is evidence this variable is related to basic need satisfaction and work outcomes (e.g. Meyer et al., 2002; Van den Broeck et al., 2008; Vansteenkiste et al., 2007) | -.03* Turnover intention | -.03* and -.15* TM practices  .14* Work engagement | .04* and .05* TM practices  .06* Work engagement |
| Oliveira et al. (2015) | N/A | Not significant | Not significant | -.21** HPWS  .15* Turnover intention |
| Oliveira & Rocha (2017) | N/A | Not significant | -.13* turnover intention | -.16** Turnover intention |
| Ramaprasad et al. (2021) | N/A | -.16* Work engagement  .14* Turnover intention | .19* Work engagement  -.18* Turnover intention | .16* Work engagement  -.30* Turnover intention |
| Rezwan & Takahashi (2021) | N/A | Not significant | .185* Retention intentions | Not significant |
| Sheehan et al. (2019) | Consistent with previous research (e.g. Holland et al., 2013) | - | -.15* Career development | Not significant |
| Sousa et al. (2021) | Relation with perceived HR practices e.g. Kooij et al., 2010), work engagement (e.g. Kim and Kang, 2017), organization commitment (e.g. Mathieu and Zajac, 1990), and turnover intention (e.g. Cotton and Tuttle, 1986) | - | -.09* Age-diversity practices  .15** Work engagement  -.36** Turnover intention | - |
| Zhong et al. (2016) | Related to job engagement (e.g., Avery, McKay, & Wilson, 2007; Rothbard, 2001; Sonnentag, 2003) | Not significant | -.08* High-Performance HR Practices  -.17** Turnover intention | -.10* High-Performance HR Practices  -.11* Turnover Intention |

**Supplementary material reference list**

Albrecht, S. L., & Marty, A. (2020). Personality, self-efficacy and job resources and their associations with employee engagement, affective commitment and turnover intentions. *International Journal of Human Resource Management,* 31(5), 657–81. <https://doi.org/10.1080/09585192.2017.1362660>

Alferaih, A., Sarwar, S., & Eid, A. (2018). Talent turnover and retention research: The case of tourism sector organisations in Saudi Arabia. *Evidence-based HRM Global Forum Empirical Scholarshi*p, 6(2), 166–86. <https://doi.org/10.1108/EBHRM-06-2017-0035>

Alfes, K., Shantz, A. D., Truss, C., & Soane, E. C. (2013). The link between perceived human resource management practices, engagement and employee behaviour: A moderated mediation model. International *Journal of Human Resource Management*, 24(2), 330–51. <https://doi.org/10.1080/09585192.2012.679950>

Alhajaj, W.E. and Ahmad, S.Z. (2023), The effectiveness of human resource management practices, work engagement and self-efficacy in minimizing talent turnover intention, *International Journal of Productivity and Performance Management,* Vol. ahead-of-print No. ahead-of-print. <https://doi.org/10.1108/IJPPM-02-2023-0090>

Ang, S. H., Bartram, T., McNeil, N., Leggat, S. G., & Stanton, P. (2013). The effects of high-performance work systems on hospital employees’ work attitudes and intention to leave: A multi-level and occupational group analysis. *International Journal of Human Resource Management*, 24(16), 3086–114. <https://doi.org/10.1080/09585192.2013.775029>

*Anjum, K., & Din, B. B. H. (2022). The role of HRM practices, work engagement, and turnover intention: The moderating effect of financial insecurity during COVID-19. *Journal of Advanced Research in Dynamical and Control Systems*, 19(2), 6768–81. <https://www.webology.org/data-cms/articles/20220328072332pmwebology%2019%20(2)%20-%20494%20pdf.pdf>

*Babakus, E., Yavas, U., & Karatepe, O. M. (2017). Work engagement and turnover intentions: Correlates and customer orientation as a moderator. *International Journal of Contemporary Hospitality Managemen*t, 29(6), 1580–98. <https://doi.org/10.1108/IJCHM-11-2015-0649>

Bui, L. T. T., & Chang, Y. (2018). Talent management and turnover intention: Focus on Danang city government in Vietnam. *International Review of Public Administration,* 23(4), 219–36. <https://doi.org/10.1080/12294659.2018.1552403>

Dalal, R., & Akdere, M. (2023). Examining the relationship between talent management and employee job‐related outcomes: The case of the Indian manufacturing industry. *Human Resource Development Quarterly*, 34(2), 201–26. <https://doi.org/10.1002/hrdq.21467>

Ehrnrooth, M., Barner-Rasmussen, W., Koveshnikov, A., & Törnroos, M. (2021). A new look at the relationships between transformational leadership and employee attitudes—Does a high‐performance work system substitute and/or enhance these relationships? *Human Resource Management,* 60(3), 377–98. <https://doi.org/10.1002/hrm.22024>

Fahmi, T. M., Abdel, H., & Mohamed, S. (2020). Examining the relationship between talent management practices, work engagement, and intention to quit of academic staff: Insights from Egyptian faculties of tourism and hotels. *International* *Journal of Hospitality and Tourism Systems,* 13. <http://www.publishingindia.com/GetBrochure.aspx?query=UERGQnJvY2h1cmVzfC81Njc0LnBkZnwvNTY3NC5wZGY=>

Gadi, P. D., & Kee, D. M. H. (2020). Workplace bullying, human resource management practices, and turnover intention: The mediating effect of work engagement: Evidence of Nigeria. *American Journal of Business*, 36(1), 62–83. <https://doi.org/10.1108/AJB-08-2020-0135>

Herr, R. M., Brokmeier, L. L., Fischer, J. E., & Mauss, D. (2022). The benefits of an employee-friendly company on job attitudes and health of employees: Findings from matched employer–employee data. *International Journal of Environmental Research and Public Health*, 19(15), 9046. <https://doi.org/10.3390/ijerph19159046>

Islam, M.S., Amin, M., Feranita, F. and Karatepe, O.M. (2023), "High-involvement work practices, work engagement and their effects on bank employees' turnover intentions: the moderating role of functional competence", *International Journal of Bank Marketing,* Vol. 41 No. 6, pp. 1360-1388. <https://doi.org/10.1108/IJBM-04-2022-0157>

*Juhdi, N., Pa’wan, F., & Hansaram, R. M. K. (2013). HR practices and turnover intention: The mediating roles of organizational commitment and organizational engagement in a selected region in Malaysia. *The International Journal of Human Resource Management*, 24(15), 3002–19. <https://doi.org/10.1080/09585192.2013.763841>

Kakkar, S., Dash, S., Vohra, N., & Saha, S. (2020). Engaging employees through effective performance management: an empirical examination. *Benchmarking: An International Journal*, 27(5), 1843–1860. <https://doi.org/10.1108/BIJ-10-2019-0440>

*Katou, A. A. (2017). How does human resource management influence organizational performance? An integrative approach-based analysis. *International Journal of Productivity and Performance Management,* 66(6), 797–821. <https://doi.org/10.1108/IJPPM-01-2016-0004>

Kim, W. (2017). Examining mediation effects of work engagement among job resources, job performance, and turnover intention. *Performance Improvement Quarterly*, 29(4), 407–425. <https://doi.org/10.1002/piq.21235>

*Kloutsiniotis, P. V., & Mihail, D. M. (2017). Linking innovative human resource practices, employee attitudes, and intention to leave in healthcare services. *Employee Relations*, 39(1), 34–53. <https://doi.org/10.1108/ER-11-2015-0205>

*Kossyva, D., Theriou, G., Aggelidis, V., Sarigiannidis, L., & Chatzoudes, D. (2021). Retention of Generation Y employees through high-performance work systems, change management, and employee engagement. *European Research Studies Journal*, XXIV(4B), 66–86. DOI: 10.35808/ersj/2632

*Kossyva, D., Theriou, G., Aggelidis, V. and Sarigiannidis, L. (2024), "Retaining talent in knowledge-intensive services: enhancing employee engagement through human resource, knowledge and change management", *Journal of Knowledge Management,* Vol. 28 No. 2, pp. 409-439. <https://doi.org/10.1108/JKM-03-2022-0174>

Lee, M. C. C., Idris, M. A., & Tuckey, M. (2019). Supervisory coaching and performance feedback as mediators of the relationships between leadership styles, work engagement, and turnover intention. *Human Resource Development International*, 22(3), 257–282. <https://doi.org/10.1080/13678868.2018.1530170>

Marescaux, E., De Winne, S., & Sels, L. (2012). HR practices and HRM outcomes: the role of basic need satisfaction. *Personnel Review*, 42(1), 4–27. <https://doi.org/10.1108/00483481311285200>

Memon, M. A., Salleh, R., & Baharom, M. N. R. (2016). The link between training satisfaction, work engagement, and turnover intention. *European Journal of Training and Development*, 40(6), 407–429. <https://doi.org/10.1108/EJTD-10-2015-0077>

*Memon, M. A., Salleh, R., & Baharom, M. N. R. (2017). The mediating role of work engagement between pay satisfaction and turnover intention. *International Journal of Economics, Management and Accounting*, 25(1), 43–69. <https://journals.iium.edu.my/enmjournal/index.php/enmj/article/view/473>

*Memon, M. A., Salleh, R., Mirza, M. Z., Cheah, J.-H., Ting, H., Ahmad, M. S., et al. (2021). Satisfaction matters: The relationships between HRM practices, work engagement and turnover intention. *International Journal of Manpower*, 42(1), 21–50. <https://doi.org/10.1108/IJM-04-2018-0127>

*Memon, M. A., Salleh, R., Mirza, M. Z., Cheah, J.-H., Ting, H., & Ahmad, M. S. (2020). Performance appraisal satisfaction and turnover intention: The mediating role of work engagement. *Management Decision*, 58(6), 1053–1066. <https://doi.org/10.1108/MD-06-2018-0685>

Oliveira, L. B. de, & Silva, F. F. R. A. da. (2015). The effects of high-performance work systems and leader-member exchange quality on employee engagement: Evidence from a Brazilian non-profit organization. *Procedia Computer Science*, 55, 1023–1030. <https://doi.org/10.1016/j.procs.2015.07.092>

*Oliveira, L. B., & Rocha, J. da C. (2017). Work engagement: Individual and situational antecedents and its relationship with turnover intention. *Revista de Administração*, 19(65), 415–431. <https://doi.org/10.7819/rbgn.v19i64.3373>

Otoo, F. N. K. (2022). Human resource development and employee turnover intentions: The mediating role of employee engagement. International *Journal of Business Ecosystem & Strategy* (2687-2293), 4(4), 1–12. <https://doi.org/10.36096/ijbes.v4i4.360>

Ramaprasad, B. S., Lakshminarayanan, S., & Pai, Y. P. (2021). Exploring the mediating role of employee attitudes in the relationship between high-performance work systems and turnover intention among IT professionals in India: A serial mediation approach. *Global Business Review*, 22(1), 197–218. <https://doi.org/10.1177/0972150918795354>

*Rezwan, R. B., & Takahashi, Y. (2022). Retention intention: does having a proactive personality matter? *Personnel Review*, 51(2), 528–542. <https://doi.org/10.1108/PR-02-2020-0073>

Rumawas, W. (2021). Talent management practices on employee turnover intention. *Journal of Theoretical and Applied Management (Jurnal Manajemen Teori dan Terapan),* 14(3), 248-263. <https://doi.org/10.20473/jmtt.v14i3.29433>

*Saks, A. M. (2006). Antecedents and consequences of employee engagement. *Journal of Managerial Psychology*, 21(7), 600–19. <https://doi.org/10.1108/02683940610690169>

Shah, S. H. A., & Beh, L.-S. (2016). Impact of motivation-enhancing practices and mediating role of talent engagement on turnover intentions: Evidence from Malaysia. *International Review of Management and Marketing*, 6(4), 823–835. <https://www.econjournals.com/index.php/irmm/article/view/3102>

*Sharma, N. P., Sharma, T., & Nanda Agarwal, M. (2022). Relationship between perceived performance management system (PMS) effectiveness, work engagement, and turnover intention: Mediation by psychological contract fulfillment. *Benchmarking*, 29(9), 2985–3007. <https://doi.org/10.1108/BIJ-01-2021-0008>

*Sheehan, C., Tham, T. L., Holland, P., & Cooper, B. (2019). Psychological contract fulfillment, engagement, and nurse professional turnover intention. *International Journal of Manpower*, 40(1), 2–16. <https://doi.org/10.1108/IJM-08-2017-0211>

Sousa, I. C., Ramos, S., & Carvalho, H. (2021). Retaining an age-diverse workforce through HRM: The mediation of work engagement and affective commitment*.* *German Journal of Human Resource Management*, 35(4), 409–435. <https://doi.org/10.1177/2397002220979797>

van den Heuvel, S., Freese, C., Schalk, R., & Van Assen, M. (2017). How change information influences attitudes toward change and turnover intention: The role of engagement, psychological contract fulfillment, and trust. *Leadership & Organization Development Journal*, 38(3), 398–418. <https://doi.org/10.1108/LODJ-03-2015-0052>

*van der Merwe, B., Malan, J., & Bruwer, R. (2020). Intention to quit in the financial services industry: Antecedents and managerial implications. *South African Journal of Economic and Management Sciences*, 23(1). DOI: 10.3390/socsci11110497

Wen, D., Yan, D., & Sun, X. (2022). Employee satisfaction, employee engagement, and turnover intention: The moderating role of position level. *Human Systems Management*, 41(3), 407–422. DOI: 10.3233/HSM-211505

*Winarno, A., Prasetio, A. P., Luturlean, B. S., & Wardhani, S. K. (2022). The link between perceived human resource practices, perceived organizational support, and employee engagement: A mediation model for turnover intention. *South African Journal of Human Resource Management*, 20(0), 7. <https://sajhrm.co.za/index.php/sajhrm/article/view/1802/2878>

Yusliza, M.-Y., Tanveer, M. I., Ramayah, T., Kumar, S. C., Saputra, J., & Noor Faezah, J. (2021). Perceived green human resource management among employees in manufacturing firms. *Polish Journal of Management Studies*, 23(1), 470–486. DOI: 10.17512/pjms.2021.23.1.29

*Zhong, L., Wayne, S. J., & Liden, R. C. (2016). Job engagement, perceived organizational support, high-performance human resource practices, and cultural value orientations: A cross-level investigation. *Journal of Organizational Behavior*, 37(6), 823–844. <https://doi.org/10.1002/job.2076>
